# POLQ inhibition elicits an immune response in homologous recombination–deficient pancreatic adenocarcinoma via cGAS/STING signaling

**DOI:** 10.1172/JCI165934

**Published:** 2023-06-01

**Authors:** Grace Oh, Annie Wang, Lidong Wang, Jiufeng Li, Gregor Werba, Daniel Weissinger, Ende Zhao, Surajit Dhara, Rosmel E. Hernandez, Amanda Ackermann, Sarina Porcella, Despoina Kalfakakou, Igor Dolgalev, Emily Kawaler, Talia Golan, Theodore H. Welling, Agnel Sfeir, Diane M. Simeone

**Affiliations:** 1 Department of Surgery and; 2 Perlmutter Cancer Center, NYU Langone Health, New York, New York, USA.; 3 Molecular Biology Program, Memorial Sloan Kettering Cancer Center, New York, New York, USA.; 4 Department of Pathology, NYU Langone Health, New York, New York, USA.; 5 Sheba University, Tel Aviv, Israel.

**Keywords:** Cell Biology, Cancer, DNA repair, T cells

## Abstract

Pancreatic ductal adenocarcinoma (PDAC) is a highly lethal malignancy that harbors mutations in homologous recombination–repair (HR-repair) proteins in 20%–25% of cases. Defects in HR impart a specific vulnerability to poly ADP ribose polymerase inhibitors and platinum-containing chemotherapy in tumor cells. However, not all patients who receive these therapies respond, and many who initially respond ultimately develop resistance. Inactivation of the HR pathway is associated with the overexpression of polymerase theta (Polθ, or POLQ). This key enzyme regulates the microhomology-mediated end-joining (MMEJ) pathway of double-strand break (DSB) repair. Using human and murine HR-deficient PDAC models, we found that POLQ knockdown is synthetically lethal in combination with mutations in HR genes such as *BRCA1* and *BRCA2* and the DNA damage repair gene *ATM*. Further, POLQ knockdown enhances cytosolic micronuclei formation and activates signaling of cyclic GMP-AMP synthase–stimulator of interferon genes (cGAS-STING), leading to enhanced infiltration of activated CD8^+^ T cells in BRCA2-deficient PDAC tumors in vivo. Overall, POLQ, a key mediator in the MMEJ pathway, is critical for DSB repair in BRCA2-deficient PDAC. Its inhibition represents a synthetic lethal approach to blocking tumor growth while concurrently activating the cGAS-STING signaling pathway to enhance tumor immune infiltration, highlighting what we believe to be a new role for POLQ in the tumor immune environment.

## Introduction

Pancreatic ductal adenocarcinoma (PDAC) is a highly lethal malignancy typically diagnosed at an advanced stage and is minimally responsive to chemotherapy and ionizing radiation. Over the past decade, large-scale whole-genome sequencing analyses have revealed a complex mutational landscape where more than 90% of PDAC tumors possess oncogenic mutations in *KRAS,* and inactivation of *TP53*, *SMAD4*, and *CDKN2A* occurs in 50%–75% of patients (1). However, mutations in additional genes occur at lower frequencies and contribute to significant intra- and intertumoral heterogeneity (1). Not surprisingly, therapeutic strategies that employ an unselective approach may have limited effectiveness. Despite such heterogeneity, a subset of patients with PDAC, constituting up to 25% of cases, possesses mutations in genes involved in DNA damage response (DDR) (1–3). Most of these mutations occur in DDR genes involved in the homologous recombination–repair (HR-repair) pathway (3). Among the mutations conferring HR deficiency (HRD), germline *BRCA1* and *BRCA2* mutations are observed in 4%–7% of all patients with PDAC (4). Notably, these genes are mutated at a frequency approaching 20% in high-risk populations. In addition, whole genome sequencing of tumors of patients with PDAC tumors suggests that an additional 7% of patients without these known mutations have features of HRD (5).

DNA damage frequently occurs due to endogenous factors (e.g., errors in replication and oxidative stress) and exogenous factors (e.g., chemotherapy and ionizing radiation). Since accurate repair of DNA breaks is crucial to maintaining genomic integrity, multiple overlapping pathways exist to repair such lesions (6). In cancer cells, the inability to repair DNA lesions promotes genomic instability and enhances the mutation rate, ultimately driving tumor evolution and progression (7, 8). Conversely, loss of function in one or more DDR genes sensitizes cells to DNA-damaging therapeutics. Mutations in *BRCA1* and *BRCA2* enhance tumor sensitivity to platinum-based chemotherapy in breast (9), ovarian (10, 11), and pancreatic cancer (12, 13).

DDR-targeting drugs have been developed based on the concept of synthetic lethality, in which a DDR mutation confers a specific vulnerability to the cancer cell after another DDR gene is targeted by a therapeutic drug. One classic example of synthetic lethality is the use of poly ADP ribose polymerase inhibitors (PARPi) to treat tumors defective in HR repair (6). Upon DNA damage, PARP1 binds to unrepaired single-strand and double-strand breaks to recruit downstream effectors (14). PARPi thereby trap PARP1 on DNA strands, leading to DNA damage that remains unrepaired (15). This promotes further genomic instability and cell death without functional HR repair (15). However, only a fraction of patients with BRCA mutations respond to PARP inhibition, and many who initially respond develop resistance, leading to cancer progression (16). Thus, new therapies that are effective in HR-deficient PDAC, alone or in combination with existing regimens, are needed.

In addition to the 2 canonical double-strand break (DSB) repair pathways, namely HR and classical nonhomologous end joining (C-NHEJ), another distinct pathway called alternative nonhomologous end joining or microhomology-mediated end joining (MMEJ) has been described (17). The MMEJ pathway competes with HR during the S phase of the cell cycle to bind DNA ends while introducing short stretches of templated insertions in the process (18). Due to its high mutagenicity, MMEJ was initially thought to act as a backup mechanism in DNA repair. However, recent studies have revealed that MMEJ operates even when HR and C-NHEJ are active and may be the only pathway to repair specific types of DNA lesions (19).

A key player in MMEJ is polymerase theta (Polθ; encoded by the POLQ gene), which mediates the joining of 2 resected 3′ ends with DNA sequence microhomology (20, 21). Although POLQ is undetectable in most normal tissues (22), it is frequently overexpressed in many cancers and correlates with a poorer prognosis (22–26). Within tumors, POLQ expression is also elevated in HRD, with synthetic lethality exhibited between POLQ depletion and mutations in genes of the HR repair pathway (27). As POLQ is crucial to the survival of HR-deficient cells, it is an emerging target in cancer therapeutics. However, the therapeutic potential for POLQ inhibition in HR-deficient PDAC has yet to be explored.

In this study, we demonstrated, for what we believe to be the first time, that POLQ is a promising therapeutic target in HR-deficient pancreatic cancer using short hairpin RNA-mediated (shRNA-mediated) downregulation in both human and murine models of HR-deficient PDAC. We found that POLQ knockdown is synthetically lethal with mutations in HR genes such as *BRCA1* and *BRCA2*, as well as the DNA damage repair gene *ATM*, and demonstrate the potential role of POLQi. Further, we show that POLQ inhibition causes the accumulation of cytosolic DNA to activate the cyclic GMP-AMP synthase–stimulator of interferon genes (cGAS-STING) signaling pathway in BRCA2-deficient pancreatic tumors, thus enhancing intratumoral T cell infiltration. In parallel, POLQ inhibition elicits synthetic lethality and synergizes with PARPi in HR-deficient PDAC cells.

## Results

### POLQ expression is elevated in HR-deficient and genomically unstable PDAC.

**Figure 1:**
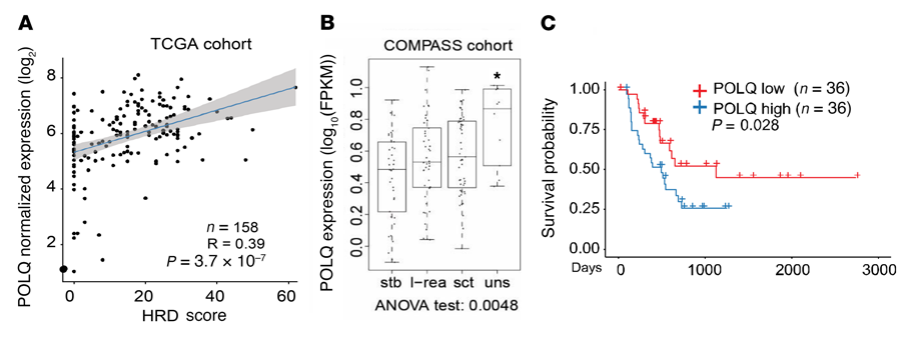
*POLQ* expression is elevated in HR-deficient PDAC. (**A**) Within the TCGA cohort, resected PDAC specimens with elevated HRD scores correlate with higher levels of *POLQ* expression. HRD, homologous recombination deficiency. (**B**) When the COMPASS cohort is subdivided by genome structure (13), genomically unstable PDAC tumors have the highest *POLQ* expression. Stb, stable; l-rea, locally rearranged; sct, scattered; uns, unstable. (**C**) In the TCGA cohort, patients with PDAC expressing higher levels of *POLQ* have poorer survival compared with patients with PDAC and lower *POLQ* levels (*P* = 0.028).

To assess the clinical significance of *POLQ* expression in PDAC, in silico analyses of existing databases were performed. Previous large-scale studies have demonstrated that up to 25% of human PDAC tumors harbor mutations conferring HRD (28) and the mutational signature captured by an HRD score (29). When 158 resected PDAC samples from the Cancer Genome Atlas (TCGA) data set were analyzed, an elevated HRD score was associated with higher *POLQ* expression (Figure 1A; Supplemental Figure 1; supplemental material available online with this article; https://doi.org/10.1172/JCI165934DS1). Waddell et al. used genomic data to identify 4 subtypes of PDAC, noting that tumors with unstable genomes harbored mutations in DDR genes (13). When applying these genomic structure groups to the cohort of patients with advanced disease in the Comprehensive Molecular Characterization of Advanced PDAC for Better Treatment Selection (COMPASS, NCT02750657) study, *POLQ* expression was highest in tumors with an unstable genomic structure (*P* = 3.78e-07, Figure 1B) (30).

To assess the clinical significance of *POLQ* expression in PDAC, we evaluated overall survival of patients with PDAC in relation to *POLQ* expression. Using the TCGA cohort, a survival plot was generated comparing samples ranked in the top and bottom 30% of *POLQ* expression. Elevated *POLQ* expression levels significantly correlated with worse survival (*P* = 0.028, Figure 1C).

### Inactivation of POLQ elicits synthetic lethality and induces DNA damage in HR-deficient PDAC.

To test our central hypothesis that POLQ inhibition blocks the growth of HR-deficient PDAC, we employed murine PDAC cell lines lacking either *BRCA1*, *BRCA2*, or *ATM* and evaluated cell proliferation and colony formation in the presence and absence of POLQ. These cell lines were established from tumors developed in genetically engineered PDAC mouse models expressing oncogenic *LSL-Kras*^G12D/+^; *LSL-Trp53*^R172H/+^; *Pdx1-Cre* (KPC), crossed with mice carrying conditional alleles of the Brca1, Brca2, or Atm genes to generate KPC*-Brca1*^–/–^, KPC*-Brca2*^–/–^, and KC*-Atm*^–/–^ mice. Because *Cre* is expressed only in the pancreatic epithelial lineage, this combination of alleles resulted in the deletion of HR and DDR genes specifically in the pancreas. Pancreatic tumors that subsequently developed resembled human PDAC deficient in HR or DDR.

**Figure 2:**
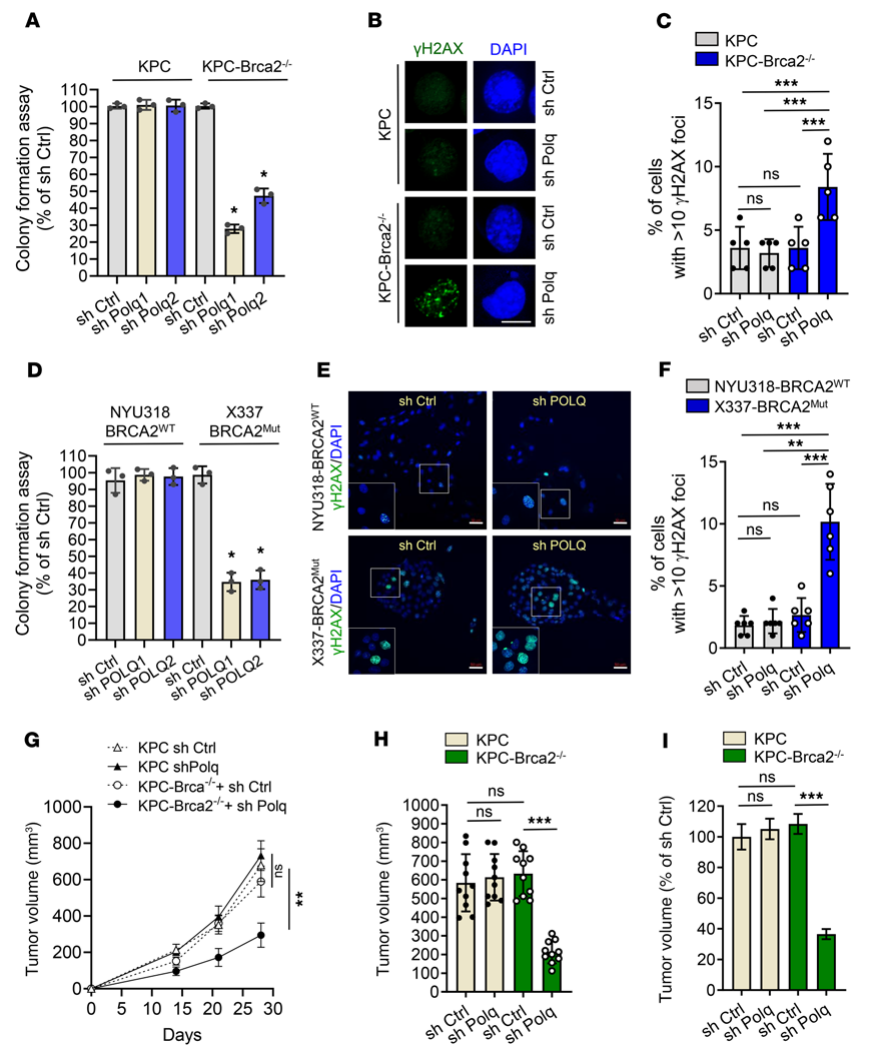
POLQ inhibition induces synthetic lethality in BRCA2-deficient PDAC. (**A**) POLQ inhibition reduces colony formation in KPC-*Brca2^–/–^* cells but does not affect colony formation in KPC cells (*n* = 3). (**B**) Representative images of immunofluorescence staining for γH2AX (green) and DAPI (blue) in KPC and KPC-*Brca2^–/–^* cells, shCtrl and shP*OLQ*. Scale bar: 10 μm. (**C**) Quantification of cells with more than 10 γH2AX foci from **B**. Each point on the graph represents 1 visual field. (**D**) POLQ inhibition reduces colony formation in X337 cells (*BRCA2*^Mut^) but has minimal effect on colony formation in NYU 318 cells (*BRCA2*^WT^) (*n* = 3). (**E**) Representative images of immunofluorescence staining for γH2AX (green) and DAPI (blue) in NYU318 and X337 cells, shCtrl and shP*OLQ*. Scale bar: 50 μm. The white box indicates the nuclei shown in the magnified inset. (**F**) Quantification of cells with more than 10 γH2AX foci from **E**. (**G**) Growth curves of KPC and KPC-*Brca2^–/–^* tumors with shCtrl or sh*Polq* (*n* = 10/group). (**H**) Tumor volume was measured by ultrasound in C57BL/6 mice orthotopically implanted with KPC and KPC-*Brca2^–/–^* shCtrl and shP*OLQ* cells (*n* = 10 mice/group). (**I**) Tumor volume from **H** as a percentage of the average tumor volume in the KPC shCtrl group (*n* = 10 mice/group). Data are representative of at least 3 independent experiments. *P < 0.05, **P < 0.01, ***P < 0.005. Error bars indicate the mean ± SEM.

Cells were transduced with control shRNA or shRNA POLQ1 or -2, targeting different regions of *POLQ*. Successful *POLQ* knockdown was verified with RT-PCR and revealed similar knockdown levels with each of the shRNAs across all cell lines (Supplemental Figure 2, A and C). POLQ knockdown significantly reduced colony formation in KPC*-Brca2^–/–^* cells (Figure 2A), with no effect in control KPC cells, confirming a strong synthetic lethal relationship between HR-deficiency and POLQ inactivation in the setting of *Kras* and *Trp53* mutations. Like KPC-*Brca2^–/–^* cells, KPC cells harboring *Brca1* mutations also showed decreased colony formation with sh*POLQ* (Supplemental Figure 2, B and C). Importantly, while *ATM* mutant tumors have been reported to be relatively insensitive to PARP inhibition, POLQ knockdown also exhibited a significant effect in Atm-deficient KPC cells (Supplemental Figure 2, B and C).

Since cells rely on POLQ-mediated DNA repair in the setting of HRD, we next examined γH2AX foci formation as a biological marker of accumulated DNA damage. POLQ knockdown increased γH2AX foci in KPC-*Brca2^–/–^* cells, while levels of γH2AX foci did not significantly differ between shCtrl and shP*OLQ* for KPC cells (Figure 2, B and C). We also performed Rad51 foci formation assays on KPC and KPC-*Brca2^–/–^* tumor cells, both untreated or in response to 10 Gy ionizing radiation (IR) (Supplemental Figure 2, D and E). In the absence of IR, no significant difference in Rad51 foci formation was observed between KPC and KPC-*Brca2^–/–^* tumors, while shRNA-mediated POLQ inhibition resulted in a small but significant elevation in Rad51 foci formation in KPC-Br*ca2^–/–^* cells but not in KPC cells. A much larger increase in Rad51 foci formation was observed in KPC mice in response to IR, further enhanced by shRNA-mediated inhibition of POLQ. However, Rad51 foci formation in KPC-*Brca2^–/–^* cells was much lower than in KPC cells in the presence of IR-induced DNA damage, with no significant difference in these cells with shRNA-mediated POLQ inhibition. These data suggest that the reduced growth in vitro as exhibited in the KPC-Brca2^–/–^ cells was, at least in part, due to faulty DNA repair mechanisms. We further tested Rad51 foci formation in ATM-deficient KC cell lines with or without POLQ inhibition to check whether the Rad51 activity is solely dependent on Brca2 or other DDR genes as well. As shown in Supplemental Figure 2, panels F and G, IR significantly increased Rad51 foci formation in both KPC and KC-*Atm*^–/–^ cells. POLQ inhibition did not affect Rad51 foci formation in KC-*Atm*^–/–^ with or without IR treatment, suggesting that Brca2 is required for Rad51 foci formation. In addition, we performed replication protein A (RPA) foci formation assays in KPC and KPC-*Brca2*^–/–^ cells. RPA foci formation was very low in nonradiated KPC and KPC-*Brca2*^–/–^ cells, and POLQ inhibition had no effect. Administration of IR significantly increased RPA formation in KPC cells, which was unaffected by Brca2 deficiency and POLQ knockdown (Supplemental Figure 2, H and I).

To assess if these observed effects of POLQ knockdown were seen in human models, shPOLQ1 and 2 were transfected into human PDAC-derived cell lines possessing mutations in various DDR genes. We observed reduced colony formation with shP*OLQ1* and 2 in X337, a *BRCA2*^S1982fs^ (*BRCA2*^Mut^) cell line, while shP*OLQ* had no significant effect on NYU318, a WT *BRCA2* (*BRCA2*^WT^) cell line (Figure 2D and Supplemental Figure 2J). Similarly, shP*OLQ* reduced colony formation in NYU228, a cell line with a pathogenic *ATM*^R3008H^ mutation (*ATM*^Mut^), but not in *ATM*-WT (*ATM*^WT^) MIAPaCa-2 cells (Supplemental Figure 2, K and L). We also examined γH2AX foci as a biological marker of accumulated DNA damage in human PDAC cell lines. POLQ knockdown increased γH2AX foci in X337-*BRCA2*^Mut^ cells, while levels of γH2AX foci did not significantly differ between shCtrl and shP*OLQ* for NYU318-*BRCA2*^WT^ cells (Figure 2, E and F).

### Effects of POLQi on HR-deficient PDAC models in vitro.

**Figure 3:**
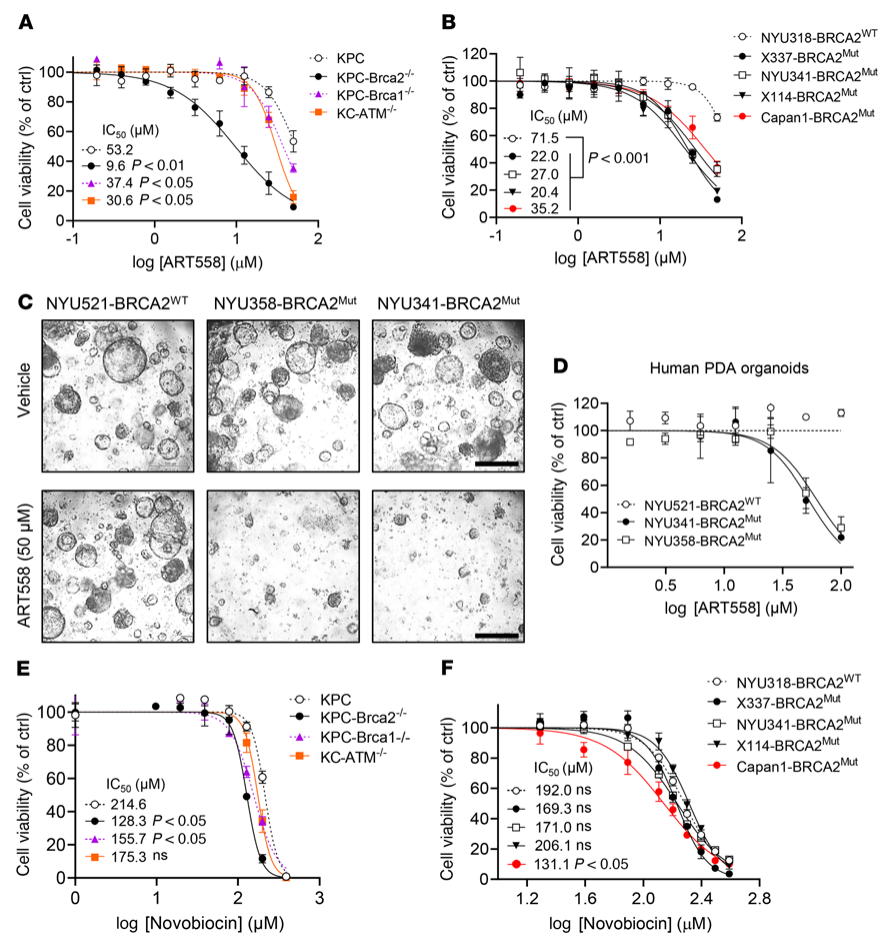
POLQi elicits synthetic lethality and synergizes with PARPi in HR-deficient PDAC cells. (**A**) Dose-dependent viability assays of KPC, KPC-*Brca2^–/–^*, KPC-*Brca1^–/–^*, and KC-*Atm*^–/–^ cells exposed to ART558 at the indicated concentrations. Cell viability was measured by CellTiter-Glo after 6 days of drug exposure. Data are displayed as cell viability relative to the control. (**B**) Dose-dependent viability assays of NYU318-*BRCA2*^WT^, X337-*BRCA2*^Mut^, NYU341-*BRCA2*^Mut^, X114-*BRCA2*^Mut^, and Capan1-*BRCA2*^Mut^ cells exposed to ART558 at the indicated concentrations. Data are displayed as cell viability relative to the control. (**C**) Representative images of human PDAC NYU521-*BRCA2*^WT^, NYU341-*BRCA2*^Mut^, and NYU358-*BRCA2*^Mut^ organoids treated with increasing concentrations of ART558 as indicated. Scale bar: 500 μm. (**D**) Dose-dependent viability assays of human PDAC organoids, NYU521-*BRCA2*^WT^, NYU341-*BRCA2*^Mut^, and NYU358-*BRCA2*^Mut^ exposed to ART558 at the indicated concentrations. Data are displayed as cell viability relative to the control. (**E**) Dose-dependent viability assays of KPC, KPC-*Brca2^–/–^*, KPC-*Brca1^–/–^* and KC-*Atm*^–/–^ cells exposed to Novobiocin (NVB) at the indicated concentrations after 6 days of drug exposure. Data are displayed as cell viability relative to the control. (**F**) Dose-dependent viability assays of NYU318-*BRCA2*^WT^, X337-*BRCA2*^Mut^, NYU 341-*BRCA2*^Mut^, X114-*BRCA2*^Mut^, and Capan1-*BRCA2*^Mut^ cells exposed to NVB at the indicated concentrations. Data are representative of at least 3 independent experiments. *P < 0.05, **P < 0.01, ***P < 0.005. Error bars indicate the mean ± SEM.

Based on our in vitro findings with POLQ knockdown, we decided to test the specificity and efficacy of 2 POLQi recently reported in the literature, ART558 and novobiocin (NVB) (31, 32). As previously reported, we also found ART558 to be effective in decreasing cell viability and colony formation in DLD1 colorectal adenocarcinoma cells with a truncating mutation in BRCA2 compared with isogenic WT DLD1 cells (Supplemental Figure 3, A and B). ART558 treatment was then performed in all of the different knockout KPC lines (Figure 3A) to check cell viability in response to the pharmacological inhibition of POLQ. ART558 was most effective in KPC-*Brca2*^–/–^ cells (IC_50_ 9.6 μM), with significant effects also seen in KPC-*Brca1*^–/–^ cells (IC_50_ 37 μM) and KC-*Atm^–/–^* cells (IC_50_ 30.6 μM) compared with KPC cells (IC_50_ 53.2 μM). ART558 also significantly shifted dose-response curves of X337-*BRCA2*^Mut^, NYU341-*BRCA2*^Mut^, X114-*BRCA2*^Mut^, and Capan1- *BRCA2*^Mut^ human PDAC cells compared with NYU318-*BRCA2*^WT^ cells (Figure 3B). Of note, the IC_50_ values for cell death were higher in human compared with murine Brca2-deficient PDAC cell lines. A similar decrease in cell viability with ART558 was observed in human NYU341-*BRCA2*^Mut^ and NYU358-*BRCA2*^Mut^ PDAC organoids compared with NYU521-*BRCA2*^WT^ PDAC organoids (Figure 3, C and D). To further verify whether ART588 affects cell viability in the ATM-deficient setting, we utilized MiaPaCa2 PDAC cells as well as their isogenic counterparts engineered to undergo knockout of the ATM gene. There was a statistically significant decrease in cell viability with ART558 treatment in *ATM*^–/–^ MiaPaCa2-*ATM* cells compared with the *ATM*^WT^ MiaPaCa2 cells (Supplemental Figure 3C, IC_50_ of 16.2 μM versus 25.4 μM, *P* < 0.05).

With the increasing use of PARPi for HR-deficient tumors, we tested the combinatorial effect of ART558 and the PARPi olaparib (ola) compared with single-agent treatment. Our analysis showed that ola treatment (2 μM) significantly sensitized human X337-*BRCA2*^Mut^ and X114-*BRCA2*^Mut^ PDAC cells to ART558. As a control, we observed no sensitization in NYU318-*BRCA2*^WT^ cells (Supplemental Figure 3D). Further, POLQ knockdown effectively sensitized mouse and human HR-deficient PDAC cells to ola treatment (Supplemental Figure 3, E and F), suggesting that inhibition of POLQ synergizes with PARPi in HR-deficient PDAC cells in vitro.

We also tested the antibiotic novobiocin, identified in a screen as a specific POLQi (32). NVB demonstrated a modest but significant decrease in viability in KPC-*Brca2*^–/–^ and KPC-*Brca1*^–/–^ cells compared with KPC cells (Figure 3E). The low-passage human PDAC cell lines X337-*BRCA2*^Mut^, NYU341-*BRCA2*^Mut^, and X114-*BRCA2*^Mut^ had no significant difference in their dose-response curves to NVB, though NYU318-*BRCA2*^WT^ cells did (Figure 3F). At the same time, we verified that Capan1-*BRCA2*^Mut^, a PDAC cell line previously reported to respond to NVB, had decreased cell viability in response to NVB compared with the *BRCA* WT control.

### Targeting POLQ inhibits BRCA-mutant tumor growth.

To investigate whether stable knockdown of POLQ would suppress in vivo growth of HR-deficient pancreatic tumors, we orthotopically implanted KPC and KPC-*Brca2*^–/–^ cells with POLQ knockdown into the pancreata of syngeneic C57BL/6J mice. KPC-*Brca2*^–/–^ shP*OLQ* tumors grew at a significantly slower rate and were much smaller compared with the KPC-*Brca2*^–/–^ shCtrl group 28 days after implantation (tumor volume 213 ± 19 mm^3^ versus 633 ± 38 mm^3^, *P* < 0.001), while POLQ knockdown did not affect KPC tumor size (Figure 2, G–I). POLQ knockdown in KPC-*Brca2*^–/–^ mutant tumors resulted in decreased proliferation, as measured by Ki67 expression (Supplemental Figure 2, M and N), and increased apoptosis, as measured by Caspase 3 [CC3] expression (Supplemental Figure 2, O and P). Altogether, these data support the synthetic-lethal function of POLQ in HR-deficient PDAC cells in vivo.

### POLQ inhibition activates the cGAS-STING pathway in BRCA2-deficient PDAC.

**Figure 4:**
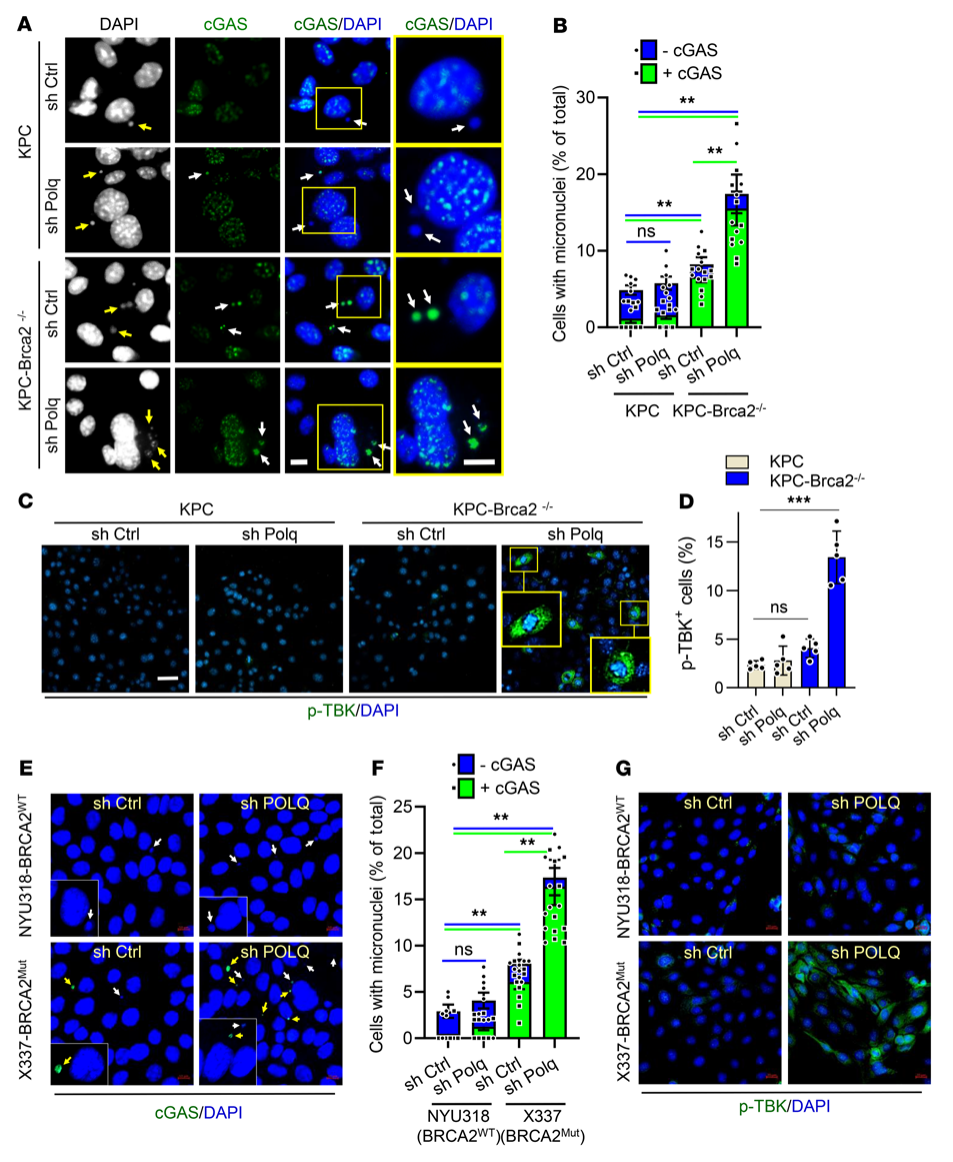
POLQ inhibition activates the cGAS-STING signaling pathway in BRCA2-deficient PDAC. (**A**) Representative images of immunofluorescence staining for cGAS (green) and DAPI (blue) in KPC and KPC-*Brca2^–/–^* shCtrl and shPOLQ cells. Arrows indicate micronuclei. The scale bar in the third column, 10 μm, applies to the first 3 columns, DAPI, cGAS, and cGAS/DAPI. Column 4, cGAS/DAPI, is a magnified view of the yellow boxes in column 3. Column 4 scale bar: 5 μm. (**B**) Quantification of cells with cGAS^+,^ cGAS^–^, and total micronuclei from **A**. Each point on the graph represents 1 visual field. (**C**) Representative images of immunofluorescence staining for p-TBK (green) and DAPI (blue) in KPC and KPC-*Brca2^–/–^* shCtrl and sh*POLQ* cells. Insets indicate magnified views of staining patterns. Scale bar: 20 μm. (**D**) Quantification of cells that are p-TBK^+^ from **C**. (**E**) Representative image of immunofluorescence staining for cGAS (green) and DAPI (blue) in NYU318^WT^ and X337-*BRCA2*^Mut^ shCtrl and shP*OLQ* cells. Scale bar: 10 μm. White arrows indicate cGAS-negative micronuclei. Arrows indicated cGAS-positive micronuclei. Insets indicate magnified views of staining patterns. (**F**) Quantification of cells with micronuclei from **E**. (**G**) Representative images of immunofluorescence staining for p-TBK (green) and DAPI (blue) in NYU318-*BRCA2*^WT^ and X337-*BRCA2*^Mut^ shCtrl and shP*OLQ* cells. Scale bar: 20 μm. Data are representative of at least 3 independent experiments. *P < 0.05, **P < 0.01, ***P < 0.005. Error bars represent the mean ± SEM.

Micronuclei (MN) are generated in the setting of genomic instability when unrepaired DNA fragments from the nucleus relocate to the cytoplasm, activating the cGAS-STING pathway (33–35). POLQ inhibition has been observed to increase micronuclei formation in cells treated with DNA-damaging agents or with mutations conferring DDR deficiency (e.g., in *FANCD2* and *BRCA1*) (21, 31, 36). Thus, we investigated how POLQ knockdown affected micronuclei formation in in vitro PDAC models by micronuclei count and cGAS staining assessment, as cGAS accumulates in micronuclei. We found that basal levels of micronuclei formation showed a small but statistically significant increase in KPC-*Brca2*^–/–^ shCtrl cells compared with KPC Ctrl cells (Figure 4, A and B), with the predominant portion of micronuclei expressing cGAS. Knockdown of POLQ resulted in a further increase in the number of cGAS-expressing micronuclei in KPC-*Brca2*^–/–^ cells, a finding not observed in WT KPC cells (Figure 4, A and B). A similar effect was observed in KPC-*Brca1*^–/–^ and KC-*Atm*^–/–^ cells (Supplemental Figure 4, A–C). As cGAS is known to participate in the activation of the STING pathway, we assessed the role of POLQ in STING signaling by investigating downstream effectors of STING. Tank-binding kinase 1 (TBK1) is phosphorylated upon STING activation and leads to subsequent phosphorylation of the transcription factor interferon regulatory factor 3 (IRF3), which induces a type I–interferon response (35, 37). Immunofluorescence analysis showed elevated p-TBK1 levels with POLQ knockdown in KPC-*Brca2*^–/–^ cells but not in KPC cells (Figure 4, C and D). Pharmacological inhibition of POLQ with ART558 mirrored the results of POLQ knockdown on micronuclei formation and p-TBK levels in these cell lines (Supplemental Figure 4, D–K).

Like KPC-*Brca2*^–/–^ cells, human PDAC X337-*BRCA2*^Mut^ shCtrl cells demonstrated significantly higher levels of both micronuclei and cGAS expression than NYU318-*BRCA2*^WT^ shCtrl cells (Figure 4, E and F). POLQ knockdown further elevated cGAS^+^ micronuclei formation in X337-*BRCA2*^Mut^ cells, an effect not observed in NUY318 shP*OLQ* cells. Downstream of cGAS-STING activation, p-TBK1 expression increased with shP*OLQ* in X337-*BRCA2*^Mut^ cells but not in NYU318-*BRCA2*^WT^ cells (Figure 4G and Supplemental Figure 4L). The addition of ola in the setting of POLQ inhibition on KPC-*Brca2*^–/–^ cells significantly upregulated the proportion of cGAS^+^MN^+^ cells (Supplemental Figure 4, M and N) and p-TBK^+^ cells (Supplemental Figure 4, O and P).

### POLQ inhibition selectively increases inflammatory cytokines in BRCA2-deficient PDAC.

The role of the cGAS-STING pathway in regulating the immune system has garnered significant interest (38). The accumulation of aberrant DNA in the cytoplasm of cancer cells can lead to STING activation, thereby inducing the expression of type I Interferons that play a critical role in innate and adaptive immune cell activation (37, 39). Recently, it has been shown that STING activation in PDAC stimulates inflammatory chemokine and cytokine expression in vivo and results in increased numbers of activated, cytotoxic CD8^+^ T cells and the reprogramming of TAMs from a M2 to M1 phenotype (40). Thus, we assessed how POLQ inhibition, with its observed effects on cGAS-STING signaling, influences the tumor immune response. First, we conducted in vitro cytokine arrays to determine the expression of cytokines previously shown to be upregulated by STING activation (34, 41). While shP*OLQ* did not change the levels of IFNγ, CXCL10, CCL5, and CXCL9 in KPC cells, these cytokines/chemokines were significantly higher in KPC-Brca2^–/–^ cells with shP*OLQ* expression compared with shCtrl (Supplemental Figure 5, A–D). Similarly, X337-*BRCA2*^Mut^ cells exhibited significantly elevated IFNγ, CXCL10, CCL5, and CXCL9 expression with shP*OLQ*, while shP*OLQ* had minimal effect on cytokine levels in NYU318-*BRCA2*^WT^ cells (Supplemental Figure 5, E–H). Further, we demonstrated upregulation of both IFNγ and CXCL10 in KPC-*Brca2*^–/–^ cells in response to POLQ knockdown and PARPi ola, and the effect was additive with POLQ knockdown and PARP inhibition combined (Supplemental Figure 5, I and J).

### POLQ inhibition alters the immune landscape and increases infiltration of activated CD8^+^ T cells in BRCA2-deficient PDAC.

**Figure 5:**
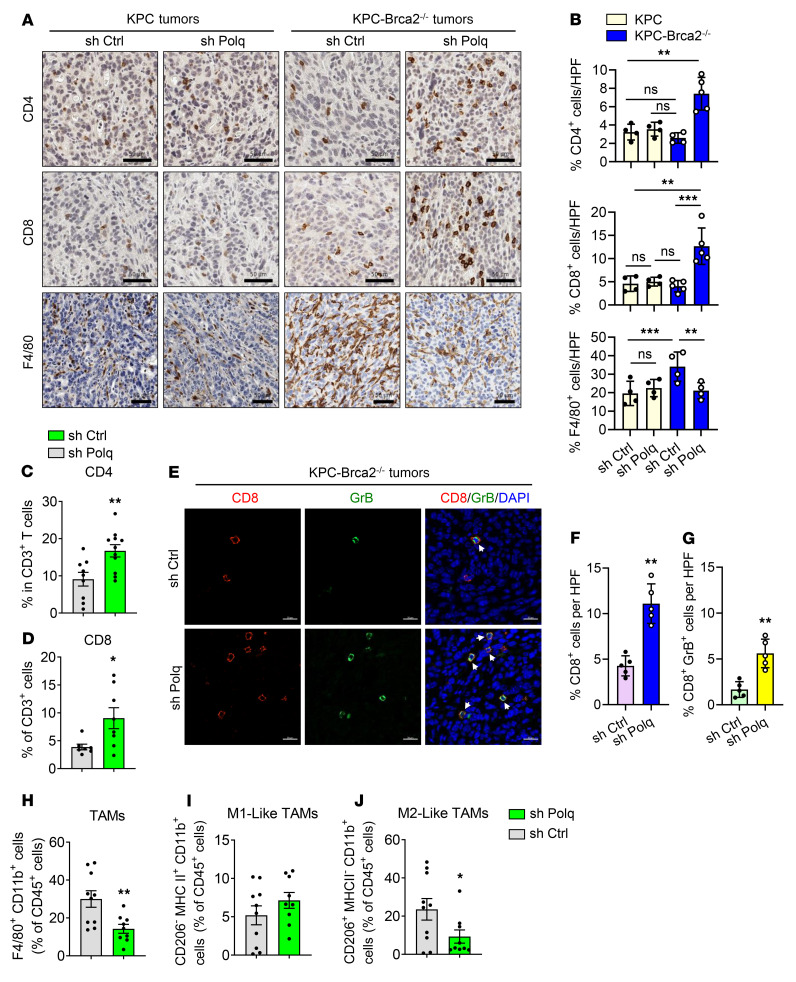
POLQ inhibition enhances immune infiltration in BRCA2-deficient PDAC. (**A**) Representative images of KPC and KPC-*Brca2^–/–^* shCtrl and shP*OLQ* tumor sections stained for CD4^+^, CD8^+^ T cells, and F4/80^+^ cells by IHC. Scale bar: 50 μm. (**B**) Quantification of CD4^+^ (upper), CD8^+^ T cells (middle), and F4/80^+^ cells (lower) from **A**. Each point on the graph represents 1 mouse (*n* = 5 mice/group). HPF, high-power field. (**C**) CD4^+^ cells as a percentage of CD3^+^ T cells during flow cytometry of KPC-*Brca2^–/–^* tumors, shCtrl, and shP*OLQ* (*n* = 10 tumors). (**D**) CD8^+^ cells as a percentage of CD3^+^ T cells during flow cytometry of KPC-*Brca2^–/–^* tumors with shCtrl and shP*OLQ* (*n* = 10 tumors). (**E**) Representative images of coIF staining of CD8 (red), Granzyme B (GrB; green), and DAPI (blue) in KPC-*Brca2^–/–^* shCtrl and shP*OLQ* tumors. Scale bar: 20 μm. (**F** and **G**) Quantification of the percent of CD8^+^ cells (**F**) or percent of CD8^+^ and GrB^+^ double-positive cells (**G**) from **E** (*n* = 5 tumors). (**H**) CD11b^+^ cells as a percentage of CD45^+^ cells during flow cytometry of KPC-*Brca2^–/–^* tumors with shCtrl and shPOLQ (*n* = 10 tumors). (**I**) CD206^–^ MHC II^+^ CD11b^+^ cells as a percentage of CD45^+^ cells during flow cytometry of KPC-*Brca2^–/–^* tumors with shCtrl, and shP*OLQ* (*n* = 10 tumors). (**J**) CD206^+^ MHC II^–^ CD11b^+^ cells as a percentage of CD45^+^ cells during flow cytometry of KPC-*Brca2^–/–^* tumors with shCtrl and shP*OLQ* (*n* = 10 tumors). Data are representative of at least 3 independent experiments. *P < 0.05, **P < 0.01, ***P < 0.005. Error bars indicate the mean ± SEM.

We investigated how these findings might translate to POLQ’s role in the adaptive immune response using our in vivo orthotopic model of KPC and KPC-*Brca2*^–/–^ shCtrl and shP*OLQ* cells, especially given that STING activation has previously been shown to affect immune cell infiltration (41–44). Tumors were harvested after 4 weeks of growth and underwent immune analysis via flow cytometry and IHC staining. *Brca2*-deficient tumors saw a significantly greater influx of CD4^+^ and CD8^+^ T cells with POLQ inhibition, whereas shP*OLQ* did not significantly affect the intratumoral CD4^+^ and CD8^+^ T cell populations in WT *Brca2* tumors (Figure 5, A–D). To assess the activation status of the infiltrated CD8^+^ T cells in the tumor microenvironment, we performed coimmunofluorescence analyses of CD8 and Granzyme B, the latter a marker indicative of activated CD8^+^ T cells (45, 46), on tumor sections. As demonstrated in Figure 5, E–G, the percentage of both CD8^+^ T cells and CD8^+^ granzyme B^+^ T cells were increased with POLQ knockdown, suggesting increased T cell–effector function in the tumor microenvironment in that setting. Of note, in addition to changes in T cell populations, KPC-*Brca2^–/–^* shP*OLQ* tumors harbored fewer tumor-associated macrophages (TAMs) than shCtrl tumors (Figure 5H). On further analysis, the decrease in TAMs with shP*OLQ* was due to a significant decrease in immunosuppressive TAMs rather than a difference in TAMs with an immune-activating phenotype (Figure 5, I and J).

### STING activation is essential for the POLQ inhibition–induced antitumor effect in BRCA2-deficient PDAC.

**Figure 6:**
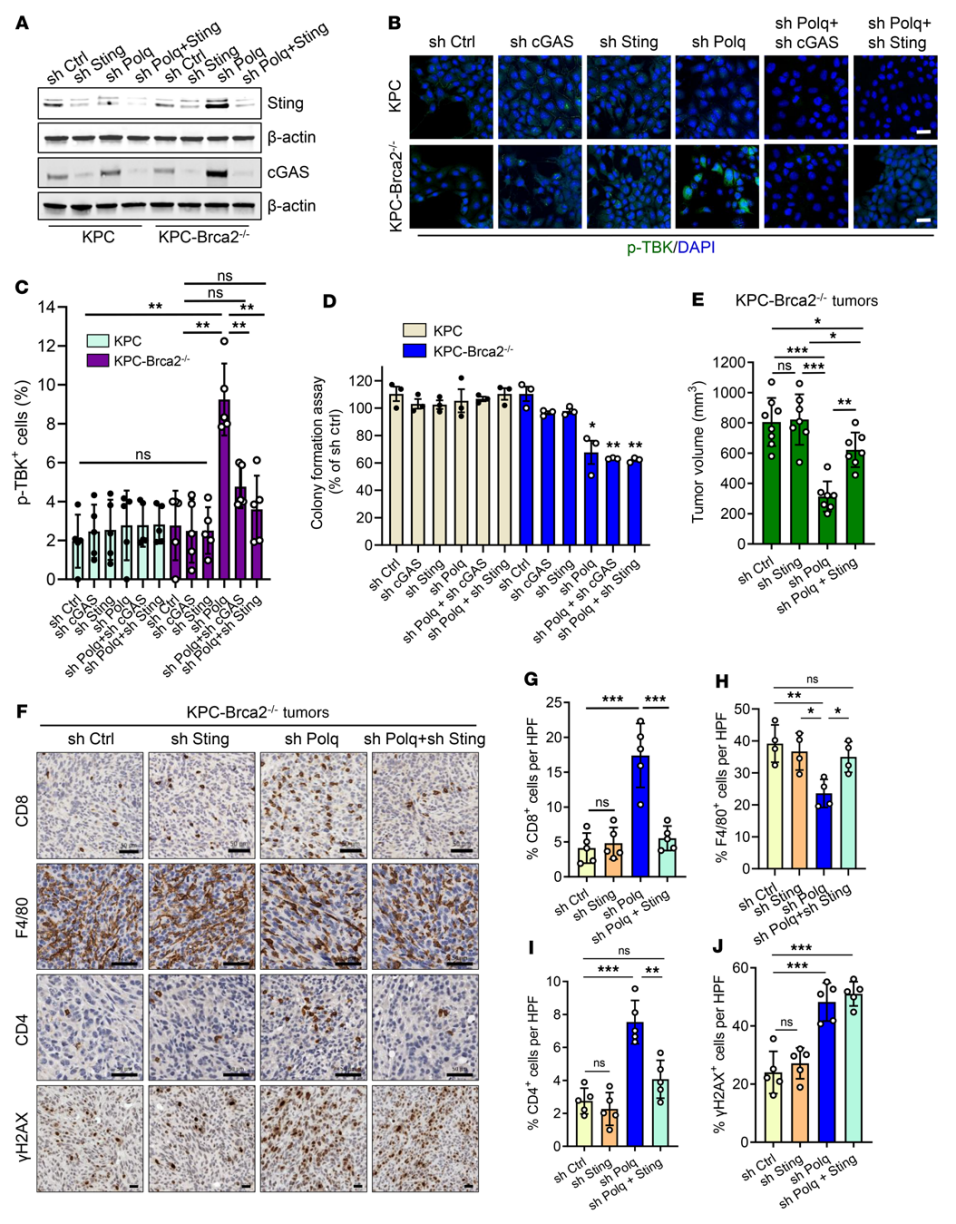
STING activation is essential for the POLQ inhibition–induced antitumor effect in BRCA2-deficient PDAC. (**A**) Western blots for cGAS and STING in KPC and KPC-*Brca2^–/–^* shCtrl with sh*CGAS*, shS*TING*, shP*OLQ*, and shP*OLQ* + shS*TING* cells. (**B**) Representative images of immunofluorescence staining for p-TBK (green) and DAPI (blue) in KPC and KPC-*Brca2^–/–^* with shCtrl, shc*GAS*, shS*TING*, shP*OLQ*, shP*OLQ* + shc*GAS*, and shP*OLQ* + shS*TING* cells. Scale bar: 10 μm. (**C**) Quantification of cells that are p-TBK^+^ from **B** (*n* = 5). (**D**) KPC-*Brca2^–/–^* shP*OLQ* cells have reduced colony formation. Additional cGAS or STING knockdown has no further effects on colony formation in KPC-*Brca2*^–/–^ cells. KPC colony formation is unaffected by shP*OLQ*, shc*GAS*, or shS*TING* (*n* = 3). (**E**) Tumor volume was measured by ultrasound in C57BL/6 mice orthotopically implanted with KPC-*Brca2^–/–^* shCtrl, shS*TING*, shP*OLQ*, and shP*OLQ* + shS*TING* tumor cells (*n* = 6 mice/group). (**F**) Representative images of KPC-*Brca2^–/–^* shCtrl, shS*TING*, shP*OLQ*, and shP*OLQ* + shS*TING* tumors stained for CD8^+^, F4/80^+^ cells, and CD4^+^ T cells and γH2AX^+^ cells by IHC. Scale bars in rows 1, 2, and 3: 50 μm. Scale bar in row 4: 20 μm. (**G**–**J**) Quantification of CD8^+^ (**G**), F4/80^+^ cells (**H**), CD4^+^ T cells (**I**), and γ–H2AX^+^ cells (**J**) from **F**. HPF, high-power field. Data are representative of at least 3 independent experiments. *P < 0.05, **P < 0.01, ***P < 0.005. Error bars indicate the mean ± SEM.

Thus far, our findings demonstrate that POLQ inhibition resulted in elevated cGAS levels in *BRCA2*-deficient PDAC models compared with WT *BRCA2* PDAC. This elevation in cGAS levels led to downstream activation of cGAS-STING signaling with increased expression of inflammatory cytokines that regulate the immune infiltrate. We next evaluated the role of cGAS and STING in the POLQ inhibition–induced immune response. We used shRNA targeting cGAS (shc*GAS*) and STING (shS*TING*) in KPC and KPC-*Brca2*^–/–^ cells and confirmed reduced cGAS and STING expression in cells with shRNA-mediated knockdown via Western blot (Figure 6A). When examining downstream STING activation, we observed that p-TBK1 levels increased more in KPC-*Brca2*^–/–^ cells with shP*OLQ* knockdown than shCtrl knockdown (Figure 6, B and C). Notably, this effect was reversed when cGAS or STING was codepleted with POLQ (Figure 6, B and C). In KPC cells, TBK1 phosphorylation remained unchanged with shP*OLQ*, whether alone or in combination with shc*GAS* or shS*TING*. Although baseline TBK1 phosphorylation was observed in cells with shc*GAS* or shS*TING*, this may be due to the fact that the shRNA-mediated knockdown system inherently does not result in total inhibition of target transcription in transfected cells. Nevertheless, inhibiting either cGAS or STING effectively prevented POLQ inhibition from further activating the pathway downstream of cGAS-STING signaling.

We next investigated the signaling pathway’s contribution to POLQ inhibition–induced growth suppression of HR-deficient PDAC. Using our in vitro models first, we observed that KPC-*Brca2*^–/–^ shc*GAS* and shS*TING* cells had similar levels of colony formation as shCtrl cells, while KPC-*Brca2*^–/–^ shP*OLQ* + shc*GAS* and shP*OLQ* + shS*TING* cells exhibited similar levels to shP*OLQ* cells (Figure 6D). Consistent with our earlier experiments, KPC-*Brca2*^–/–^ shP*OLQ* cells had decreased colony formation compared with KPC-*Brca2*^–/–^ shCtrl cells. KPC shCtrl and shP*OLQ* cells also exhibited similar levels of colony formation as in earlier experiments. Inhibition of cGAS and STING in KPC cells had no significant effect on colony growth. Based on these results, we concluded that the synthetic lethality by which POLQ and BRCA2 suppress tumor cells is a distinct mechanism independent of cGAS and STING.

Next, we used an orthotopic model to determine if STING and the resulting enhanced immune response contribute to POLQ inhibition–induced antitumor effects in *BRCA2*-deficient PDAC. We injected KPC-*Brca2*^–/–^ shCtrl, shS*TING*, shP*OLQ*, and shP*OLQ* + shS*TING* cells into the pancreata of syngeneic mice. At 4 weeks after implantation, KPC-*Brca2*^–/–^ tumors expressing shP*OLQ* were much smaller than those expressing shCtrl (shP*OLQ* 38.9% ± 4.68% of shCtrl, *P* < 0.001), while shS*TING* resulted in no significant size difference relative to shCtrl tumors (Figure 6E and Supplemental Figure 6, A–C). Notably, inhibiting STING in addition to POLQ in KPC-*Brca2*^–/–^ partially rescued the tumor size compared with the shCtrl and shS*TING* groups (shP*OLQ* + shS*TING* 77.1% ± 5.35% of shCtrl, *P* < 0.05) (Figure 6E and Supplemental Figure 6, A–C). This supports STING’s role in orchestrating the antitumor immune effects of POLQ inhibition in BRCA2-deficient PDAC. Upon tissue analysis, levels of γH2AX, indicating DNA damage, and CC3, indicating apoptosis, were similar among KPC-*Brca2*^–/–^ shCtrl and shS*TING* tumors but higher in shPOLQ and shP*OLQ* + shS*TING* tumors (Figure 6, F and J and Supplemental Figure 6, D and F). However, levels of intratumoral CD8^+^ T cells mirrored the resulting tumor size, as KPC-*Brca2*^–/–^ shP*OLQ* tumors had significantly more CD8^+^ T cell infiltration than shCtrl, shSTING, and shP*OLQ* + shS*TING* tumors (Figure 6, F–I). This suggests that STING signaling activation is critical for the POLQ inhibition–induced immune response. Ki67 decreased in shP*OLQ* tumors and was partially rescued in shP*OLQ* + shS*TING* tumors (Supplemental Figure 6, D and E), whereas elevated CC3 in shP*OLQ* tumors was not altered by shS*TING* (Supplemental Figure 6, D and F). Altogether, this indicates that POLQ inhibition induced DNA damage to ultimately trigger an influx of CD8^+^ T cells to suppress tumor growth in BRCA2-deficient PDAC, and STING represents a critical component of this pathway.

Recent findings indicate that antigen-presenting cells of the immune system also experience STING pathway activation resulting from tumor DNA and that this cross-primes CD8^+^ T cells for an antitumor response (47, 48). To further examine the contribution of STING signaling in recruited immune cells, we injected KPC-*Brca2*^–/–^ shCtrl and shP*OLQ* cells into the pancreata of *STING*^+/+^ and *STING*^–/–^ mice. At 3 weeks after implantation, KPC-Brca2^–/–^ shCtrl tumors had similar volumes in *STING*^+/+^ and *STING*^–/–^ mice, while KPC-*Brca2*^–/–^ shP*OLQ* tumors were smaller, consistent with our previous experiments (Supplemental Figure 6, G and H). However, KPC-*Brca2*^–/–^ shP*OLQ* tumors did not exhibit a significant difference in size in *STING*^+/+^ compared with *STING*^–/–^ mice (Supplemental Figure 6, G and H). On further tissue analysis, cell proliferation and apoptosis were dependent on POLQ status rather than extratumoral STING expression (Supplemental Figure 6, I–L). Likewise, CD8^+^ and F4/80^+^ cell infiltrates were similar in *STING*^+/+^ and *STING*^–/–^ mice with KPC-*Brca2*^–/–^ shP*OLQ* tumors (Supplemental Figure 6, M–P).

While our data show that STING is required for tumor immune microenvironment changes and inhibition of tumor growth, we sought to determine if there was a contribution of the adaptive immune system to the antitumor phenotype observed with POLQ deficiency. To examine this, we tested tumor growth of KPC-*Brca2*^–/–^ cells expressing either control shRNA or POLQ shRNA orthotopically injected into the pancreas of NSG immunocompromised mice. We observed no significant reduction of tumor growth, demonstrating that in vivo tumor growth mediated by the STING pathway is dependent on the tumor-immune component of the PDAC microenvironment (Supplemental Figure 6, Q–S).

Overall, these data support that, in addition to synthetic lethality between POLQ and BRCA2, cytosolic DNA damage products that accumulate in the presence of POLQ inhibition activate cGAS-STING signaling in BRCA2-deficient pancreatic cancer cells. Activation of this pathway causes transcription of downstream inflammatory cytokines and correlates with an influx of immune cells into the tumor. Moreover, STING activation is critical in this immune recruitment and in POLQ inhibition’s antitumor effect in BRCA2-deficient PDAC (Supplemental Figure 6T).

## Discussion

In our findings, POLQ is overexpressed in HR-deficient and genomically unstable PDAC and is associated with a poorer prognosis. In human and murine PDAC models, we found that POLQ inhibition is synthetically lethal with mutations in *BRCA1*, *BRCA2*, and *ATM*. We also observed increased cytosolic micronuclei formation in HR-deficient pancreatic cancer cells with POLQ inhibition and subsequent activation of the cGAS-STING pathway. This enhanced cGAS-STING signaling and increased intratumoral immune infiltration in an in vivo orthotopic model. Thus, we demonstrate for the first time that POLQ is a promising target in HR-deficient pancreatic cancer and its role in eliciting cGAS-STING signaling.

Given its low-fidelity, mutation-prone repair of double-strand breaks, POLQ has been observed to increase genomic instability in cancer cells (21, 24, 49). Additionally, POLQ represents a specific vulnerability in tumors with mutations in DDR genes, particularly genes involved in HR DNA repair, and reduces cell viability in HR-deficient cells derived from breast and ovarian tumors (24, 27, 49, 50). This revealed a synthetic lethal interaction between POLQ and HR. Here, we extend the POLQ-HR synthetic lethal interaction to pancreatic cancer cells and report a synthetic lethal interaction between ATM and POLQ. The ATM kinase pathway is predominant in response to DSBs. However, repair by NHEJ is diminished in the absence of ATM. While the mechanistic basis for the synthetic lethality of POLQ and ATM remains to be fully understood, with the well-established lethality between POLQ and NHEJ factors, including Ku and Lig4, we favor the hypothesis that the ATM and POLQ lethality is inherent to ATM’s role in promoting DSB repair. Given that up to 26% of all patients with PDAC harbor a mutation involved in HR repair or an elevated HRD score, a significant number of them may potentially benefit from targeting POLQ (3, 5).

In the setting of increased DNA damage, we also observed elevated micronuclei upon POLQ inhibition in BRCA2-deficient PDAC cells, consistent with previous reports of DNA damage leading to micronuclei formation and cGAS activation (33–35). We found that cGAS subsequently activated STING and its canonical downstream signaling pathway involving p-TBK1. Previously, other DNA-damaging agents such as radiation and etoposide have been shown to produce cytosolic DNA and enhance activation of the cGAS-STING pathway in cells, and PARP inhibition upregulates cGAS-STING signaling specifically in HR-deficient cancer cells (34, 51, 52). One study suggested that POLQ inhibition and FANCD2 deficiency activate cGAS-STING in esophageal squamous cell carcinoma (36).

To test the clinical implication of our findings, we examined the specificity and efficacy of 2 POLQi recently reported in the literature, ART558 and NVB (31, 32). Our data show that ART558 elicits synthetic lethality and synergizes with PARPi in HR-deficient PDAC cells, supporting its therapeutic potential. However, experiments preclude the use of in vivo mouse models, given that the current drug formulation is metabolized by the murine liver. On the other hand, NVB had more limited effects on cell viability in HR-deficient than in HR-proficient models. As POLQi are under evaluation for clinical trials, these findings indicate that further investigation of POLQi in specific clinical subpopulations is necessary. Of note, the data overall highlight that BRCA2-deficient cells respond more strongly to POLQi compared with BRCA1 or ATM-deficient cells in terms of dependency. Our findings present what is, to our knowledge, the first evidence for POLQ inhibition activating cGAS-STING signaling in HR-deficient cancer.

In line with our findings of cGAS-STING activation with POLQ knockdown, we found upregulation of cGAS-STING-associated inflammatory cytokines in BRCA2-deficient human and mouse cell lines. CXCL9, CXCL10, CCL5, and INFγ were among the upregulated cyto- and chemokines in our study. While CXCL9 and CXCL10 are well known for CD8^+^ T cell recruitment, a recent study in mismatch repair–deficient colorectal cancer found that recruitment and activation of CD8^+^ T cells depended on overexpression of CCL5 and CXCL10 via activation of cGAS-STING and type I Interferon signaling by damaged DNA (53). Moreover, IFNγ is a well-established M1 polarization stimulus with the potential to reprogram TAMs (54).

To investigate the relevance of POLQ knockdown–dependent cGAS-STING activation in BRCA-deficient tumors on immune cells in vivo, we employed an orthotopic model using syngeneic mice. Parallel to the increase in T cell recruitment chemokines with POLQ knockdown, we found an increase in activated CD8^+^ T cell and CD4^+^ T cell infiltrate in vivo.

Moreover, we found a decrease in overall TAMs within BRCA2-deficient tumors with POLQ inhibition. This was driven by a significant decrease in M2-like TAMs, while M1-like TAMs trended toward an increase with POLQ knockdown, although the latter was not significant. Recently, STING activation in PDAC was shown to decrease overall TAMs and reprogram M2-like to M1-like TAMs, potentially explaining a role for the effect of POLQ knockdown and subsequent STING activation on TAM polarization in our BRCA-deficient mouse model (55). While the overall decrease in TAMs cannot be explained by the cGAS-STING-associated inflammatory cytokines upregulated in our study, it is well known that IFNγ is a strong M1 polarization stimulus and might explain the trend toward M1-like TAMs. Moreover, STING activation has been shown to correlate with an immune-activating TAM phenotype in breast cancer, squamous cell carcinoma, colon cancer, and melanoma (56). In addition, BRCA1-deficient breast cancer, non–small cell lung cancer, and colitis mouse models suggest that STING activation can repolarize immunosuppressive M2-like macrophages to the immune-activating M1-like phenotype (57–59). STING’s effect on TAM repolarization may be another potential reason for the fewer immunosuppressive TAMs with POLQ knockdown in BRCA2-deficient tumors. In addition, the decrease in tumor growth with POLQ knockdown could be partly explained by antitumor immunity driven by CD8^+^ T cell influx. Moreover, STING signaling may promote macrophages and dendritic cells, both antigen presenting cells, to facilitate antigen presentation against the tumor while simultaneously decreasing immunosuppressive TAMs (48).

Although our findings demonstrate that STING is critical in the antitumor effects of POLQ inhibition in our model, STING activation has also been suggested to play a role in immune evasion in cancer (38, 43, 60). Thus, the interactions between cGAS-STING signaling and other immune cell populations, including CD4^+^ T cells, still need further investigation. Importantly, we show that the effects of POLQ in inducing microenvironmental change and tumor growth inhibition through STING signaling require an adaptive immune response, as these POLQ-mediated effects were lost in immunodeficient mice. Of note, the differences in STING expression and function in cancer cells and the tumor microenvironment suggest that the role of STING is likely variable and may depend on the type of cancer.

Nevertheless, we show that POLQ knockdown results in cGAS-STING activation and plays a complex role in the immune response that results in decreased tumor growth. Currently, STING agonists have garnered significant interest, and clinical trials are investigating their efficacy in cancer. Thus, our findings suggest that these pharmacologic therapeutics might have great potential in BRCA-deficient PDAC (61).

Overall, our findings present what is, to our knowledge, the first evidence for POLQ as a therapeutic target in HR-deficient PDAC. POLQ is synthetically lethal with several genes involved in HR repair and the DDR gene ATM. POLQ inhibition in HR-deficient PDAC also results in more cytosolic micronuclei, activating the cGAS-STING signaling pathway, enhancing transcription of inflammatory cytokines, and increasing immune infiltration in the tumor. As both POLQi and STING agonists are currently under development, our findings demonstrate a synergy between these 2 targets representing what we believe is a promising strategy to combine DNA damage repair and immunotherapy against HR-deficient pancreatic cancer.

## Methods

### Cell lines and Reagents.

The *LSL-Kras*^G12D/+^; *LSL-Trp53*^R172H/+^; *Pdx1-Cre* mouse model-derived (KPC) cell line was gifted from Dafna Bar-Sagi (NYU Langone Medical Center) (62). The KPC-*Brca2*^fl/fl^ (KPC-*Brca2*^–/–^) and KPC-*Brca1*^–/–^ cell line were gifted by Thomas Ludwig (The Ohio State Comprehensive Cancer Center, Columbus, Ohio, USA) and KC-*Atm*^–/–^ from Beatriz Sosa-Pineda (Northwestern University Feinberg School of Medicine, Chicago, Illinois, USA). Cells were cultured in RPMI-1640 (Corning) supplemented with 10% FBS.

BRCA2-mutated primary human pancreatic cancer cell lines X337 (*BRCA2*^S1982fs^) and X114 (*BRCA2*^S1982fs^), human *BRCA2* mutant PDAC cell lines, were gifted from Talia Golan (Sheba Medical Center). The parental MiaPaCa human PDAC cells and MiaPaCa2 cells with CRISPR knockout of *ATM* (MiaPaCa2-*ATM*^KO^) were obtained from Alexander Kleger (Ulm University Hospital, Ulm, Germany). The primary tumor-derived cell lines and the isogenic DLD1 cells with or without a truncating mutation in *BRCA2* (DLD1-WT and DLD1-*BRCA2*^–/–^) cells were cultured in RPMI-1640 supplemented with 10% FBS. MIA PaCa-2 was obtained from ATCC. MIA PaCa-2 was cultured in DMEM (Corning) supplemented with 10% FBS. All cell lines were additionally cultured with 100 units/mL penicillin and 100 μg/mL streptomycin (Invitrogen) and were incubated in 5% carbon dioxide at 37 °C. NVB (S2492) was purchased from Selleckchem. ART558 (HY-141520) was purchased from MedChemExpress LLC.

### Creation of isogenic cell lines.

Both human and mouse cell lines were transduced with control shRNA or shRNA targeting POLQ. shRNA for stable POLQ knockdown was purchased from Sigma (human sh*Polq*1 TRCN0000290547, sh*Polq*2 TRCN0000290548; murine sh*Polq*1 TRCN0000120313, sh*Polq*2 TRCN0000120314). Cells that underwent successful knockdown were selected by puromycin resistance (6 μg/mL in human cell lines and 10 μg/mL in mouse cell lines). Knockdown of cGAS and STING in KPC and KPC-*Brca2*^–/–^ cells was also achieved using shRNA purchased from Sigma (shc*GAS*1 TRCN0000416658, shc*GAS*2 TRCN0000178625; shS*ting*1 TRCN0000346320, sh*Sting*2 TRCN0000346266) and were selected by geneticin at 1 mg/mL (Thermo Fisher Scientific).

### Animal studies.

Experiments were conducted using 8-to-12–week old female C57BL/6 mice purchased from the Jackson Laboratories. Sting^–/–^ mice were obtained from the Jackson Laboratories (strain number 017537). Immunodeficient NSG mice were purchased from the Jackson Laboratories (strain number 005557). Syngeneic cell lines were implanted orthotopically at 200,000 cells per mouse or 500,000 cells per mouse, per specific experiment. Tumor growth was monitored weekly via ultrasound measurements. Animals were then sacrificed, and tumor weight was measured. Primary tumors were harvested and formalin-fixed for further histological analysis.

### Colony formation assays.

Cell lines with and without POLQ knockdown were plated in 6-well plates (3,000 cells/well) and incubated in 5% carbon dioxide at 37°C. After 7 days, cells were fixed in a PBS solution containing 4% paraformaldehyde. They were stained with 0.2% crystal violet and imaged. Densitometry measurements were used to determine relative colony formation.

### CellTiter-Glo cell viability assays.

Briefly, 300–2,000 cells per well were seeded in a 96-well plate in a volume of 100 μL medium. After 24 hours, cells were treated with the indicated drugs by adding 10 μL medium containing 10 × concentrated drug of the desired final concentration. Cells were cultured in the drug-containing medium for 6 days. Cell viability was determined by measuring the luminescent signal using the CellTiter-Glo luminescent cell viability assays (Promega) following the manufacturer’s instructions. The CellTiter-Glo assay determines the number of viable cells in culture based on the quantitation of the ATP present, an indicator of metabolically active cells. The surviving fraction of drug-treated cells was normalized to values from the DMSO-treated control. Survival and IC_50_ values were determined using GraphPad Prism software.

### Quantitative real-time PCR.

Quantitative real-time PCR (qRT-PCR) was performed as previously described (63). RNA was extracted from cells using the RNeasy Plus Mini Kit (Qiagen) according to the manufacturer’s instructions. cDNA was generated from RNA using the High-Capacity cDNA Reverse Transcription Kit (Life Technologies). qRT-PCR was performed using the Applied Biosystems ViiA 7 Real-Time PCR System along with TaqMan probes (Applied Biosystems) (Supplemental Table 1). PCR was conducted according to the following protocol: denaturation at 95°C for 2 minutes, 40 cycles at 95°C for 15 seconds, 60°C for 1 minute, and 72°C for 15 seconds. To verify the specificity of the PCR amplification products, a melting curve analysis was performed. mRNA levels were normalized to GAPDH or β-actin.

### IHC and immunofluorescence analysis.

Mouse KPC and KCP-*Brca2*^–/–^ cells, primary human pancreatic adenocarcinoma cell lines, or KPC and KPC-*Brca2*^–/–^ tumor sections with or without shCtrl and shP*OLQ* were fixed with 4% paraformaldehyde in PBS pH 7.4 for 15 minutes at room temperature and permeabilized with PBS containing 0.1%–0.25% Triton X-100. Routine H&E, IHC, and immunofluorescence staining were performed as previously described (63). For a list of the antibodies used, see Supplemental Table 1. Images were taken with a Panoramic DESK 3DHistech slide scanner (Caliper Life Sciences) and Nikon A1 Eclipse Ti confocal microscope. Cell nuclei were counterstained with DAPI (Life Technologies). Images were quantified using the QuPath 0.2.3 Image Analysis Software Platform.

### Western blotting.

Western blot analysis and coimmunoprecipitation experiments were performed as previously described (64). The primary antibodies used and their dilutions are listed in Supplemental Table 1. After incubation with IRDye secondary antibodies (LI-COR Biotechnology) suitable for multi-color detection, protein bands were visualized and quantified using an Odyssey CLx imager (LI-COR Biotechnology). After analysis, blots were stripped, washed, and reprobed with β-actin antibody (Sigma-Aldrich) which served as a loading control. Protein expression was quantified using Odyssey Imaging Systems Software 5.2.

### Cytokine arrays.

To detect the levels of cytokines and chemokines, KPC and KPC-*Brca2*^–/–^ cells and human NYU318-*BRCA2*^WT^, and X337-*BRCA2*^Mut^ cells were cultured for 48 hours. The culture medium was analyzed using either Mouse Cytokine Antibody Array kits or Human Cytokine Antibody Array kits (Abcam) according to the manufacturer’s instructions and analyzed on a Luminex 200 machine.

### Flow cytometry analysis.

Cell suspensions from KPC-*Brca2*^–/–^ shCtrl and shPOLQ tumors were prepared using the Mouse Tumor Dissociation Kit (Miltenyi Biotec) according to the manufacturer’s recommendations. Single cells from tumor digestion were incubated with 2 μg/mL anti-mouse CD3 (BD Biosciences, 553057), 1 μg/mL anti-mouse CD28 (BD Biosciences, 553294), and 1 μL/mL Cell Stimulation Cocktail (eBioscience) for 4 hours at 37°C in RPMI with 10% FBS. Cells were stained extracellularly with specific antibodies against mouse CD3 (557596), CD4 (560782), CD8 (563046), CD45 (560510), CD11c (563048), CD11b (562127), CD69 (553237), Ly6C (561237), F4/80 (565853), Ly6G (130-119-902), all from Miltenyi Biotec, and CK19 (ab192643), from Abcam. After washing, cells were fixed and permeabilized with Fixation/Permeabilization solution (eBioscience) and stained intracellularly with anti-IFN-γ (BD Biosciences, 561040) and anti-granzyme B (BioLegend, 515405). Samples were acquired on a flow cytometry analyzer (Attune NxT; Thermo Fisher Scientific). Data were analyzed with FlowJo software (BD Biosciences).

### TCGA and COMPASS patient data.

TCGA pancreatic cancer mRNA data underwent batch-effects normalization and was analyzed. Curated clinical data were also retrieved from the UCSC Xena Pan-Cancer Atlas hub. Survival was estimated via Kaplan-Meier curves, and significance was evaluated by log-rank tests using the “survival” R package.

### Statistics.

Data are presented as mean ± SEM. Experiments have been repeated a minimum of 3 times to demonstrate reproducibility. Statistical significance was determined by Student’s *t* test or 1-way ANOVA using GraphPad Prism 8 (GraphPad Software). *P* < 0.05 was considered significant.

### Study approval.

This study (R01CA245005) was approved by the IRB of the NIH. All animal studies were approved by the NYU Langone Health IACUC under protocol number 17-00511.

## Author contributions

DMS conceived and supervised the study. DMS, AS, GO, AW, LW, JL, GW, DW, EZ, REH, and AA designed and performed the experiments. SP, DK, ID, and EK performed the bioinformatic analyses. Additional data analysis was performed by THW and TG. GO, AW, LW, DW, and SD wrote the first draft of the manuscript, which was reviewed and edited by all of the authors. The final manuscript was approved by DMS and AS. GO is listed first in the order of co–first authors because she finished writing the manuscript.

## Supplementary Material

Supplemental data

Supplemental table 1

## Version 1 03/28/2023

In-Press Preview

## Version 2 06/01/2023

Electronic publication
