# Supplementary material for: POLQ inhibition elicits an immune response in homologous recombination–deficient pancreatic adenocarcinoma via cGAS/STING signaling
Source: J Clin Invest. 2023 Jun 1;133(11):e165934. doi: 10.1172/JCI165934 (PMC10232002; doi:10.1172/JCI165934)
Supplement: Supplemental data [file jci-133-165934-s220.pdf]

Supplemental Figure S1, related to figure 1

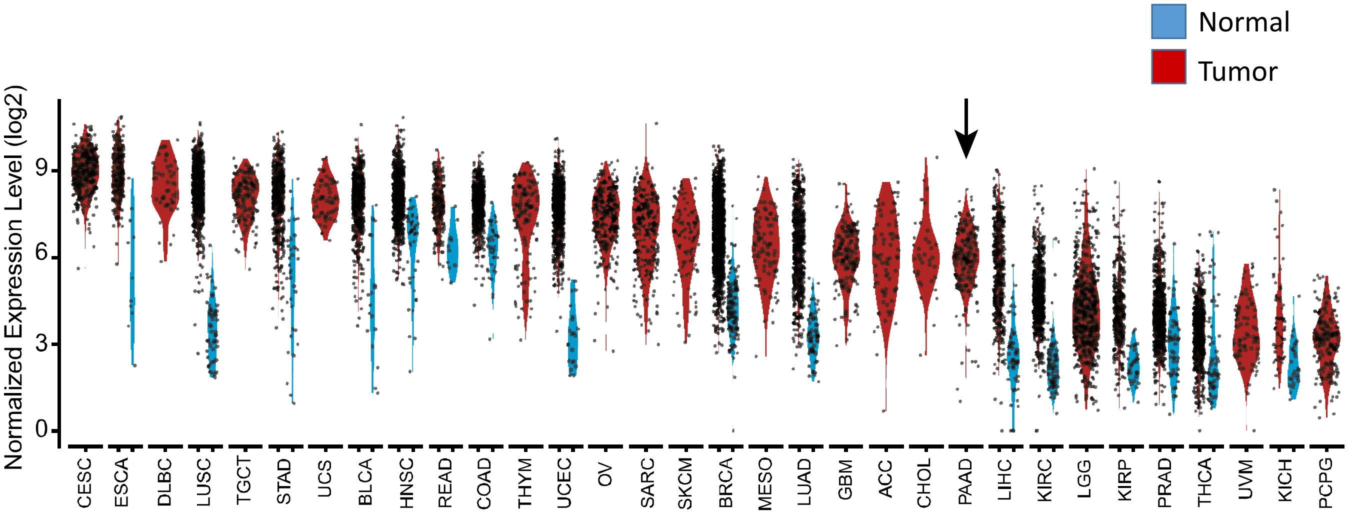

Supplemental Figure 1. *POLQ* expression is elevated in HR-deficient PDAC. Within the TCGA database, pancreatic adenocarcinoma (PAAD) (arrow) demonstrates elevated *POLQ* expression levels.

# Supplemental Figure S2, related to figure 2

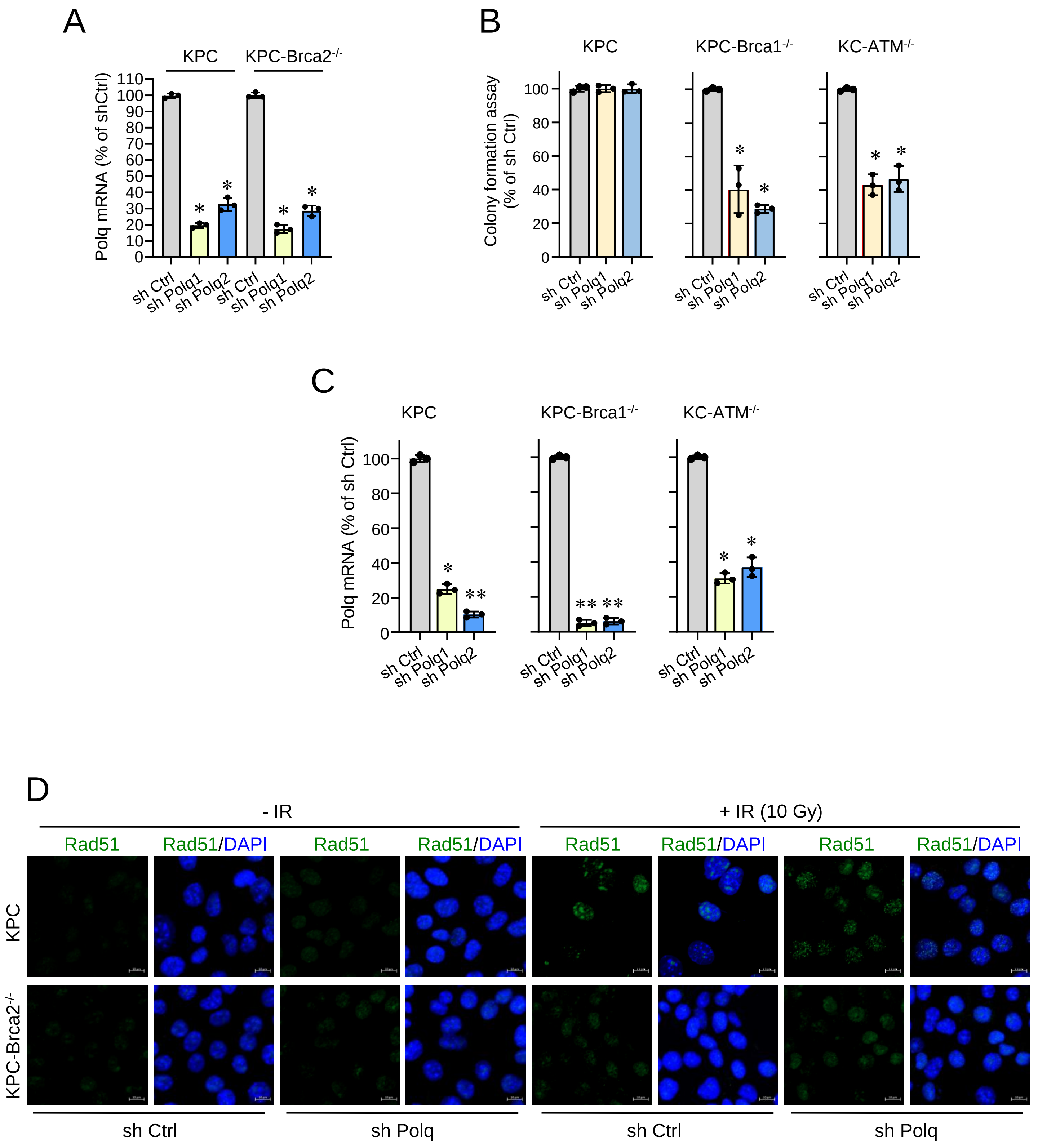

Supplemental Figure S2, related to figure 2

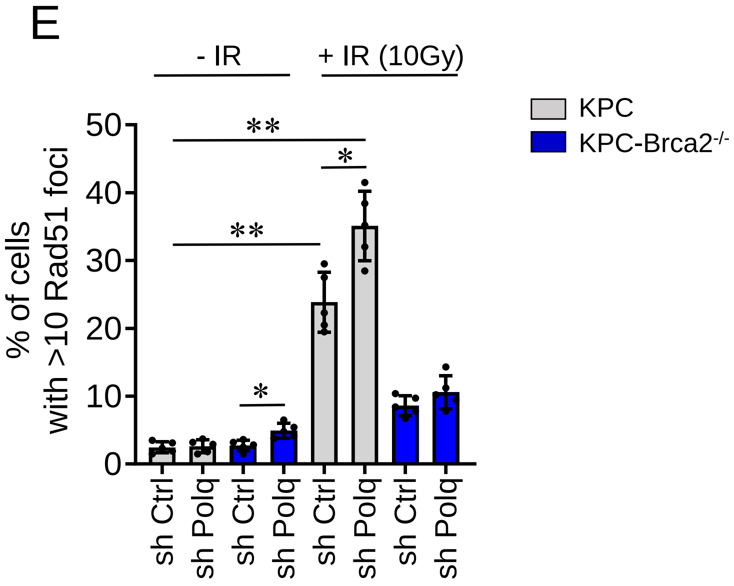


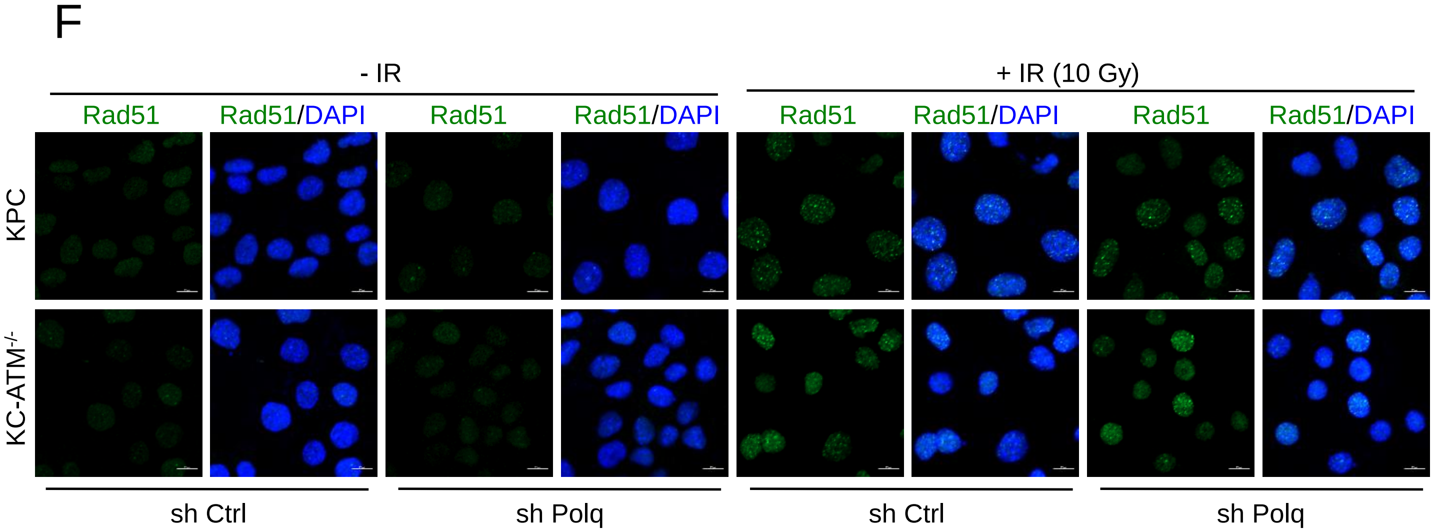


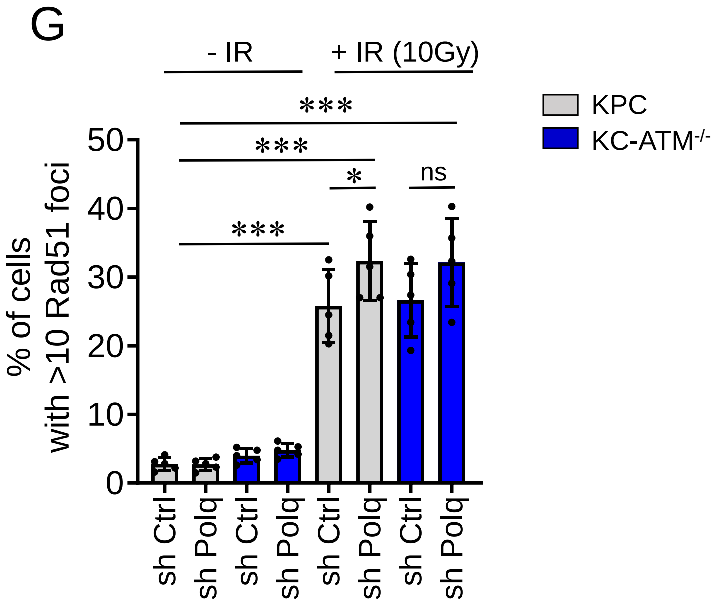

Supplemental Figure S2, related to figure 2

H

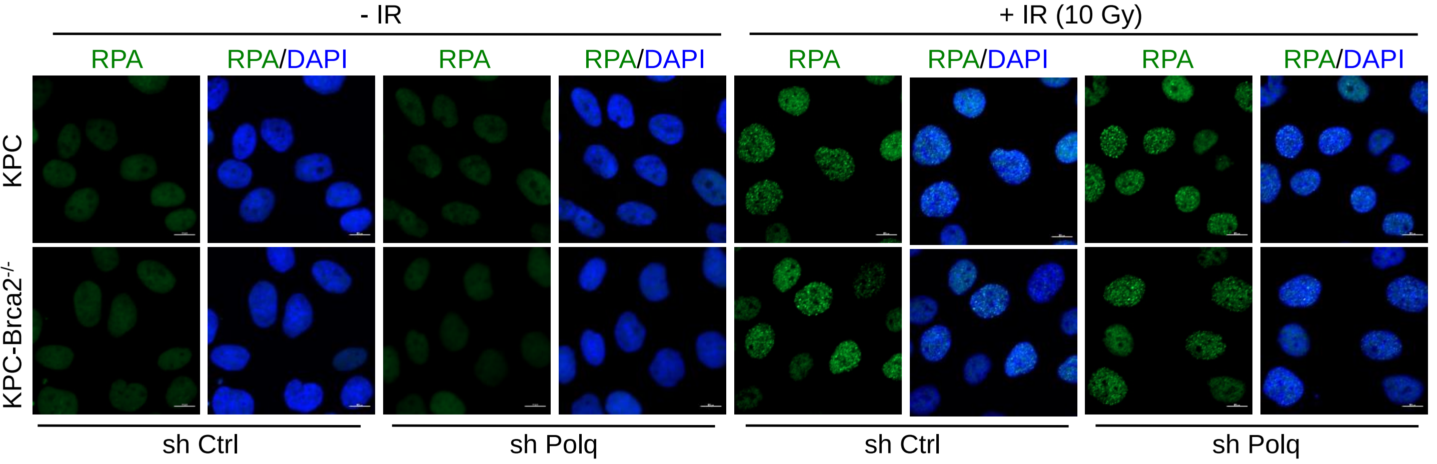


I

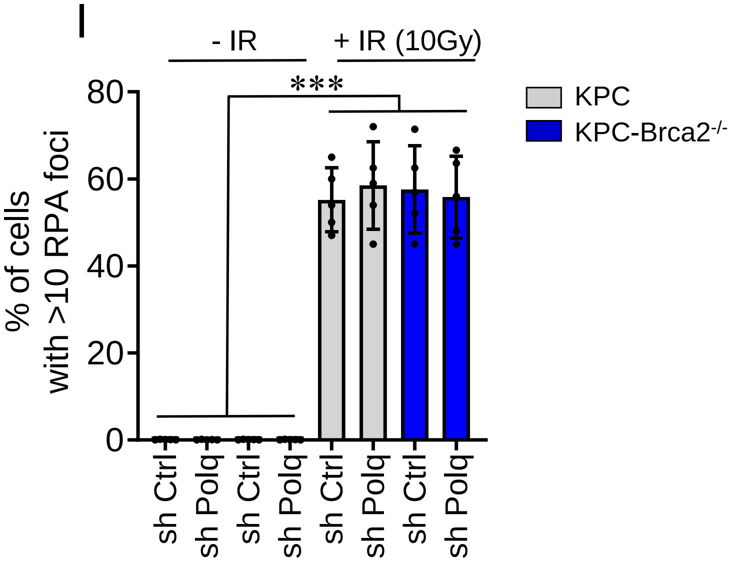


J

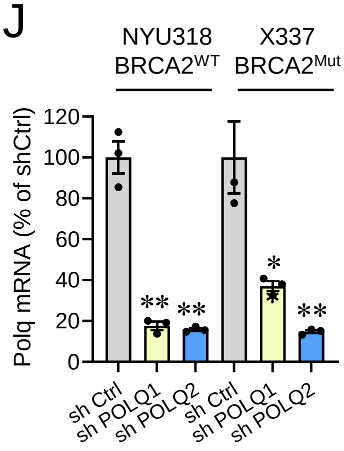


K

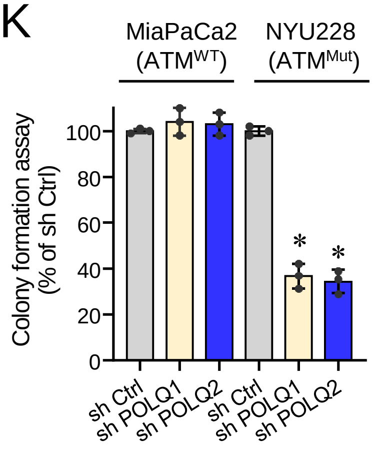


L

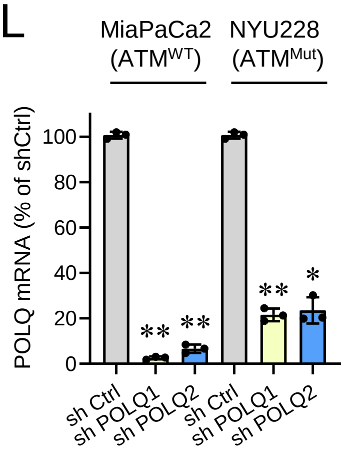

Supplemental Figure S2, related to figure 2

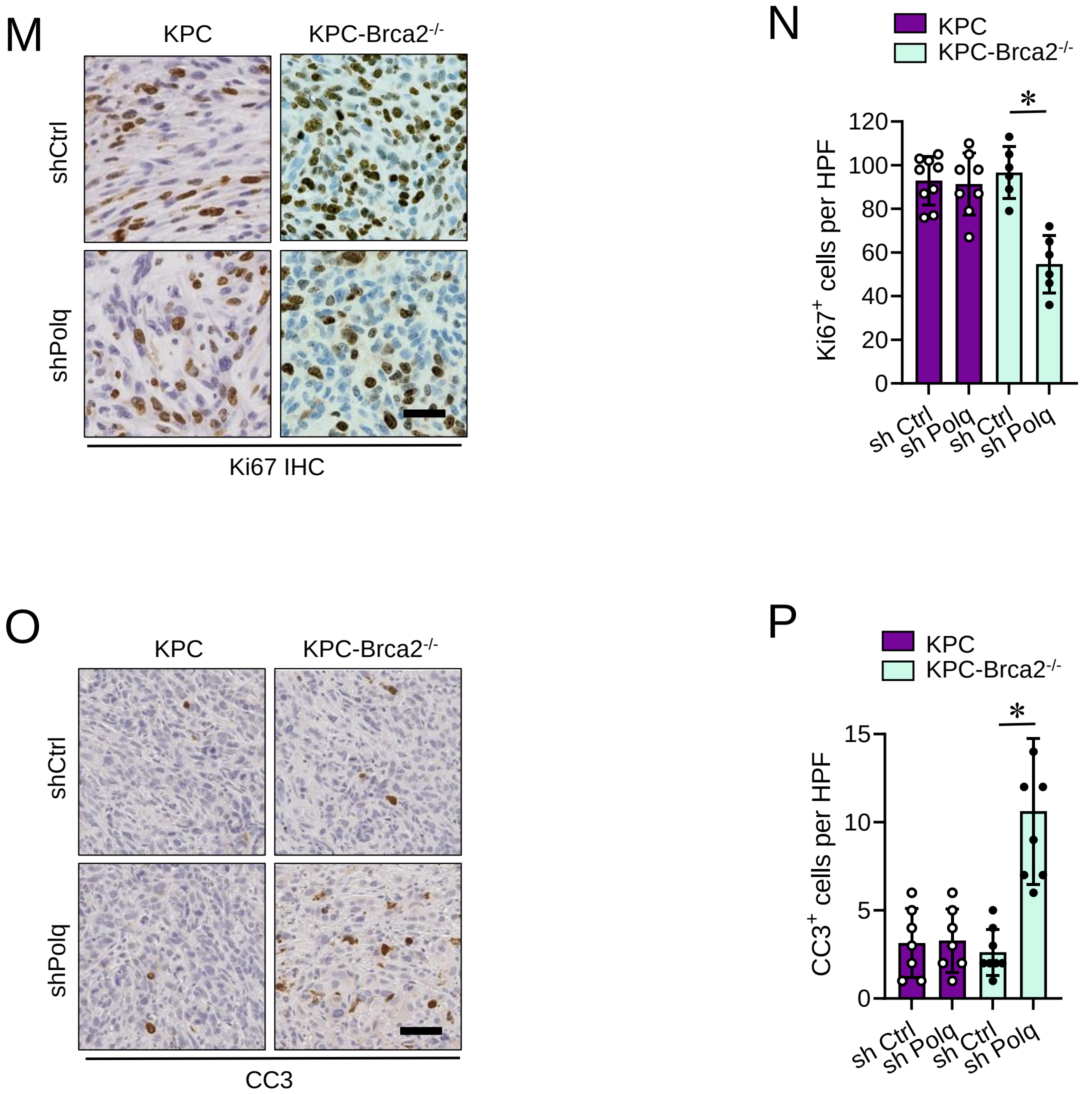

Supplemental Figure 2. POLQ inhibition induces synthetic lethality in BRCA2-deficient PDAC. (A) *POLQ* mRNA transcript levels by qRT-PCR in KPC and KPC-*Brca2*$^{-/-}$ sh Ctrl and sh POLQ cells (n=3). (B) POLQ inhibition reduces colony formation in KPC, KPC-*Brca1*$^{-/-}$ and KC-*Atm*$^{-/-}$ cell lines (n=3). (C) *POLQ* mRNA transcript levels by qRT-PCR in KPC, KPC-*Brca1*$^{-/-}$ and KC-*Atm*$^{-/-}$, cell lines (n=3). (D) Representative images of immunofluorescence staining for RAD51 (green) and DAPI (blue) in KPC and KPC-*Brca2*$^{-/-}$ sh Ctrl and sh POLQ cells. Scale bar, 10 µm. (E) Quantification of cells with more than 10 RAD51 foci from (D). Each point on the graph represents one visual field (n=5). (F) Representative images of immunofluorescence staining for RAD51 (green) and DAPI (blue) in KPC and KC-*Atm*$^{-/-}$ sh Ctrl and sh *POLQ* cells. Scale bar, 10 µm. (G) Quantification of cells with more than 10 RAD51 foci from (F). Each point on the graph represents one visual field (n=5). (H) Representative images of immunofluorescence staining for RPA (green) and DAPI (blue) in KPC and KPC-*Brca2*$^{-/-}$ sh Ctrl and sh POLQ cells. Scale bar, 10 µm. (I) Quantification of cells with more than 10 RPA foci from (H). Each point on the graph represents one visual field (n=5). (J) *POLQ* mRNA transcript levels by qRT-PCR in NYU318$^{WT}$, X337-*BRCA2*$^{Mut}$ sh Ctrl and sh *POLQ* cells (n=3). (K) POLQ inhibition reduces colony formation in NYU 228 (*ATM*$^{Mut}$) cells but has minimal effect on colony formation in MIA PaCa-2 cells (n=3). (L) *POLQ* mRNA transcript levels by qRT-PCR in MIA PaCa-2 (*ATM* WT) and NYU 228 (*ATM*$^{Mut}$) sh Ctrl and sh *POLQ* cells (n=3). (M) Representative images of tumor sections stained for Ki67 by IHC. Scale bar, 20 µm. (N) Quantification of Ki67+ cells per HPF on tumor sections stained for Ki67 by IHC (M). Each point on the graph represents one mouse (n=6-9 mice/group). (O) Representative images of tumor sections stained for CC3 by IHC. Scale bar, 50 µm. (P) Quantification of CC3+ cells per HPF on tumor sections stained for CC3 by IHC (O) (n=7-8 mice/group).

Data are representative of at least three independent experiments. (*) $P < 0.05$, (**) $P < 0.01$, (***) $P < 0.005$), (ns) not significant. Error bars=Mean ± SEM.

# Supplemental Figure S3, related to figure 3

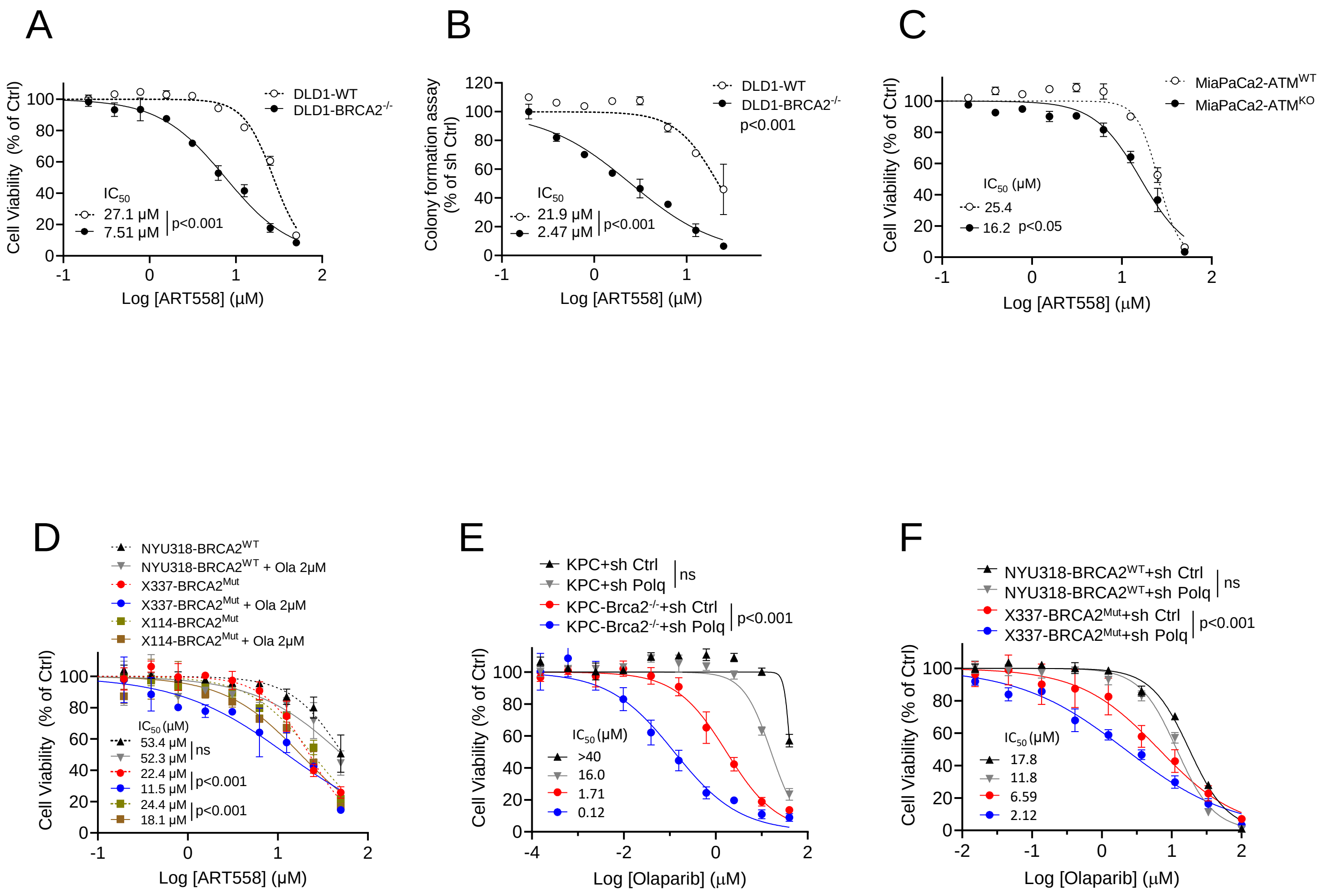

Supplemental Figure 3. POLQ inhibitor elicits synthetic lethality and synergizes with PARPi in HR-deficient PDAC cells. (A) Dose-dependent viability assays of DLD1-WT and DLD1-*BRCA2*$^{-/-}$ cells exposed to ART558 at the indicated concentrations. Cell viability was measured by CellTiter-Glo after 6 days of drug exposure. Data are % of DMSO control (% of Ctrl)(n=3). (B) Clonogenic survival of DLD1-WT and DLD1-*BRCA2*$^{-/-}$ cells with increasing concentrations of ART558. Survival is normalized to the values from the untreated sample (n=3). (C) Dose-dependent viability assays of MiaPaCa2-*ATM*$^{WT}$ and MiaPaCa2-*ATM*$^{KO}$ cells exposed to ART558 at the indicated concentrations. Cell viability was measured by CellTiter-Glo after 6 days of drug exposure. Data are % of DMSO control (% of Ctrl)(n=3). (D) Dose-dependent viability assays of NY318$^{WT}$, X337-*BRCA2*$^{Mut}$, and X114-*BRCA2*$^{Mut}$ cells exposed to increasing concentration of ART558 alone or in combination with 2 μM PARPi (Olaparib). Data are % of DMSO control (% of Ctrl). (E) Dose-dependent viability assays of KPC or KPC-*Brca2*$^{-/-}$ cells with or without sh Control (Ctrl) or sh Polq exposed to increasing concentration of Olaparib. Data are % of DMSO control (% of Ctrl). (F) Dose-dependent viability assays of NYU318-*BRCA2*$^{WT}$ or X337-*BRCA2*$^{Mut}$ cells with or without sh Control (Ctrl) or sh *Polq* exposed to increasing concentration of Olaparib. A two-way ANOVA test was used to calculate p values. Data are representative of at least three independent experiments. (*) $P < 0.05$, (**) $P < 0.01$, (***) $P < 0.005$), (ns) not significant. Error bars=Mean ± SEM.

# Supplemental Figure S4, related to figure 4

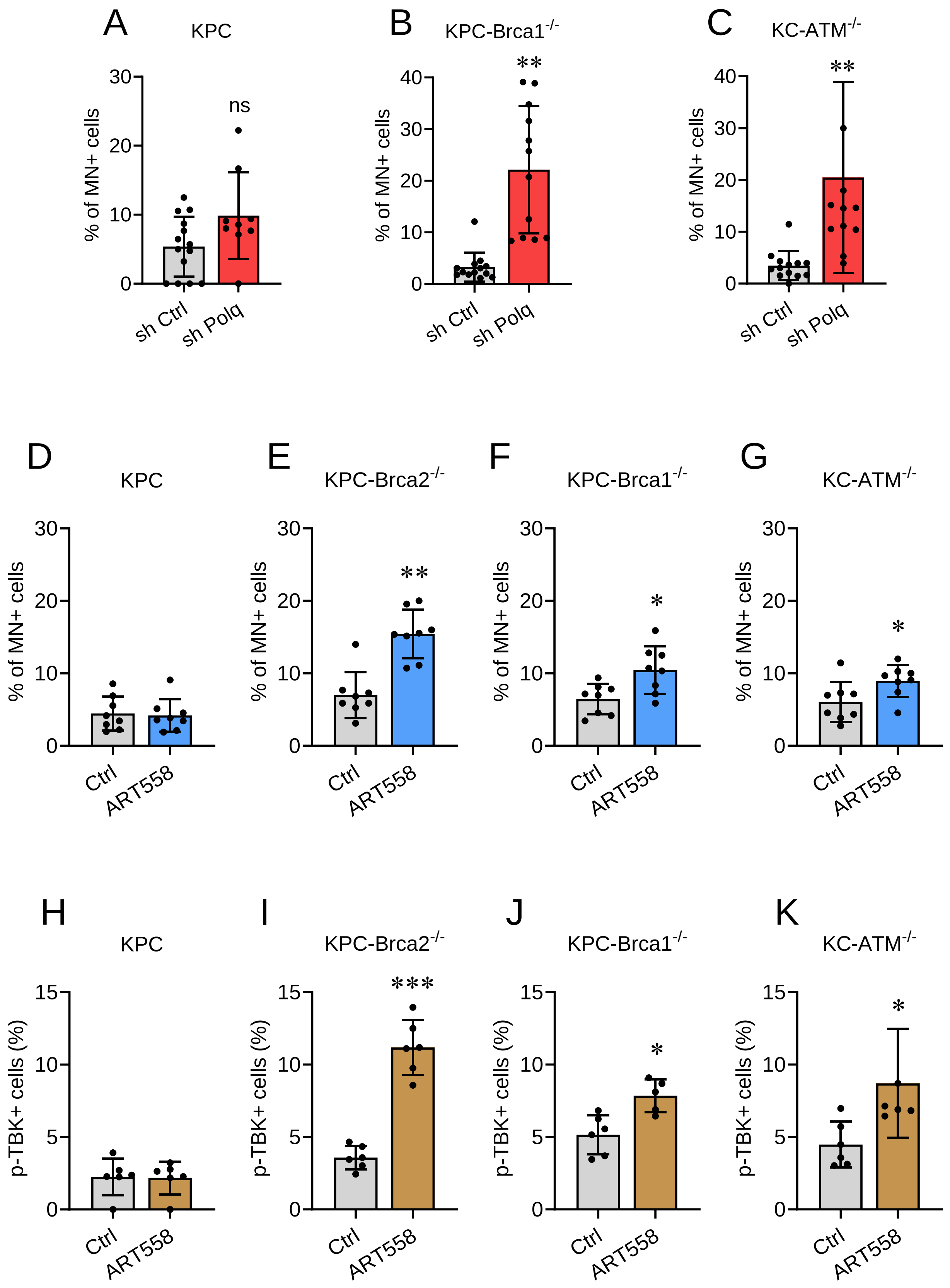

## Supplemental Figure S4, related to figure 4

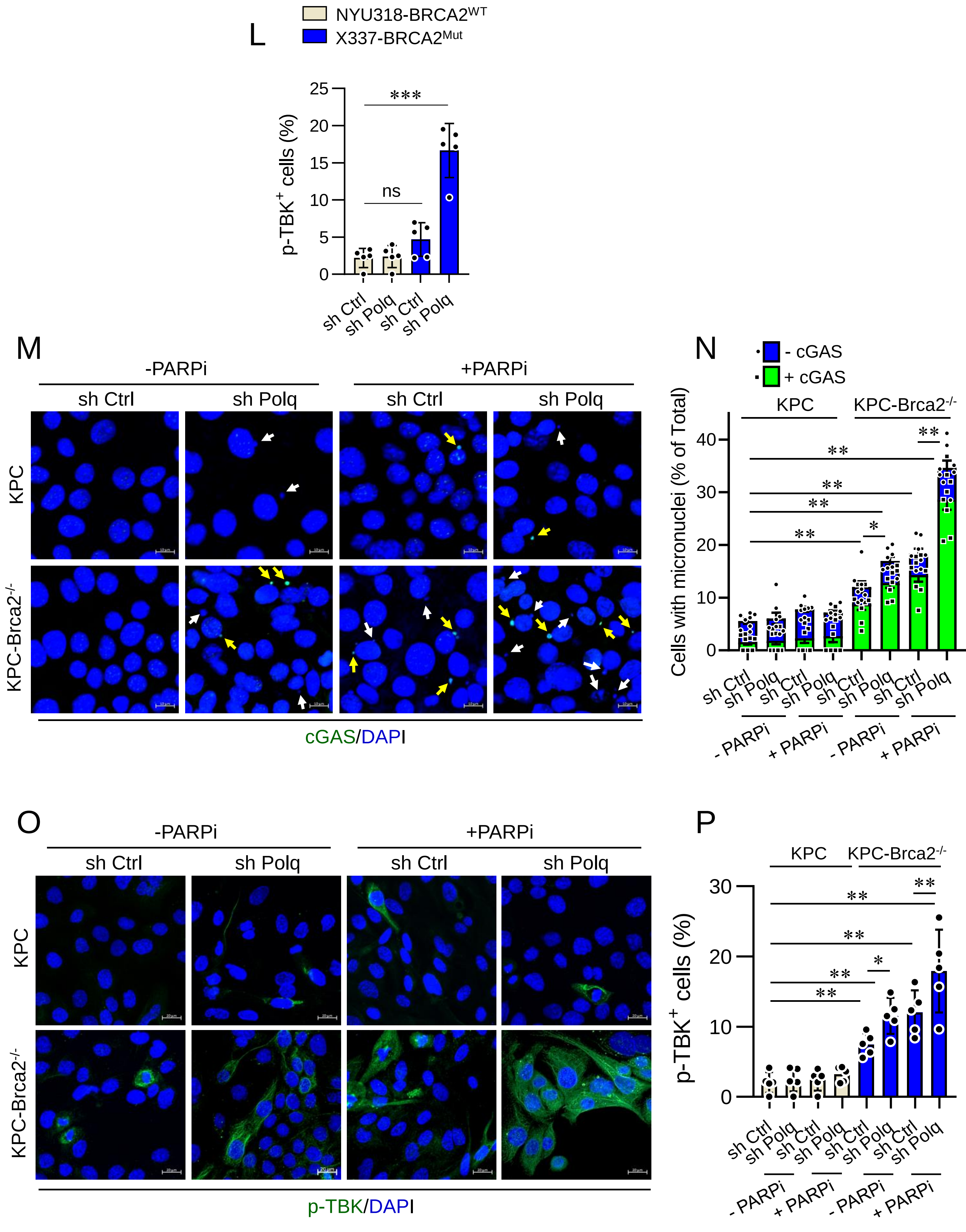

Supplemental Figure 4. POLQ inhibition activates the cGAS-STING signaling pathway in BRCA2-deficient PDAC

(A-C) Quantification of cells with micronuclei (MN) in KPC, KPC-*Brca1*$^{-/-}$, KC-*Atm*$^{-/-}$, and KPC-*Palb2*$^{-/-}$ cells with or without sh Polq expression. Each point on the graph represents one visual field. (D-G) Quantification of cells with micronuclei (MN) in KPC (D), KPC-*Brca2*$^{-/-}$ (E), KPC-*Brca1*$^{-/-}$ (F), and KC-*Atm*$^{-/-}$ (G) cells with or without POLQ inhibitor ART558 treatment. Each point on the graph represents one visual field. (H-K) Quantification of cells that are p-TBK+ in KPC (H), KPC-*Brca2*$^{-/-}$ (I), KPC-*Brca1*$^{-/-}$ (J), and KC-*Atm*$^{-/-}$ (K) cells with or without POLQ inhibitor ART558 treatment. Each point on the graph represents one visual field. (L) Quantification of cells that are p-TBK+ from (Figure 4G). (M) Representative image of immunofluorescence staining for cGAS (green) and DAPI (blue) in KPC and KPC-*Brca2*$^{-/-}$ cells with or without sh POLQ in combination with 2 µM PARPi (Olaparib). Scale bar, 10 µm. White arrows indicate cGAS cGAS-negative micronuclei. Yellow arrows indicated cGAS-positive micronuclei. (N) Quantification of cells with micronuclei from (M). (O) Representative image of immunofluorescence staining for p-TBK (green) and DAPI (blue) in KPC and KPC-*Brca2*$^{-/-}$ cells with or without sh POLQ in combination with 2 µM PARPi (Olaparib). Scale bar, 20 µm. (P) Quantification of cells that are p-TBK+ from (O).

Data are representative of at least three independent experiments. (*) $P < 0.05$, (**) $P < 0.01$, (***) $P < 0.005$), (ns) not significant. Error bars=Mean ± SEM.

Supplemental Figure S5, related to figure 5

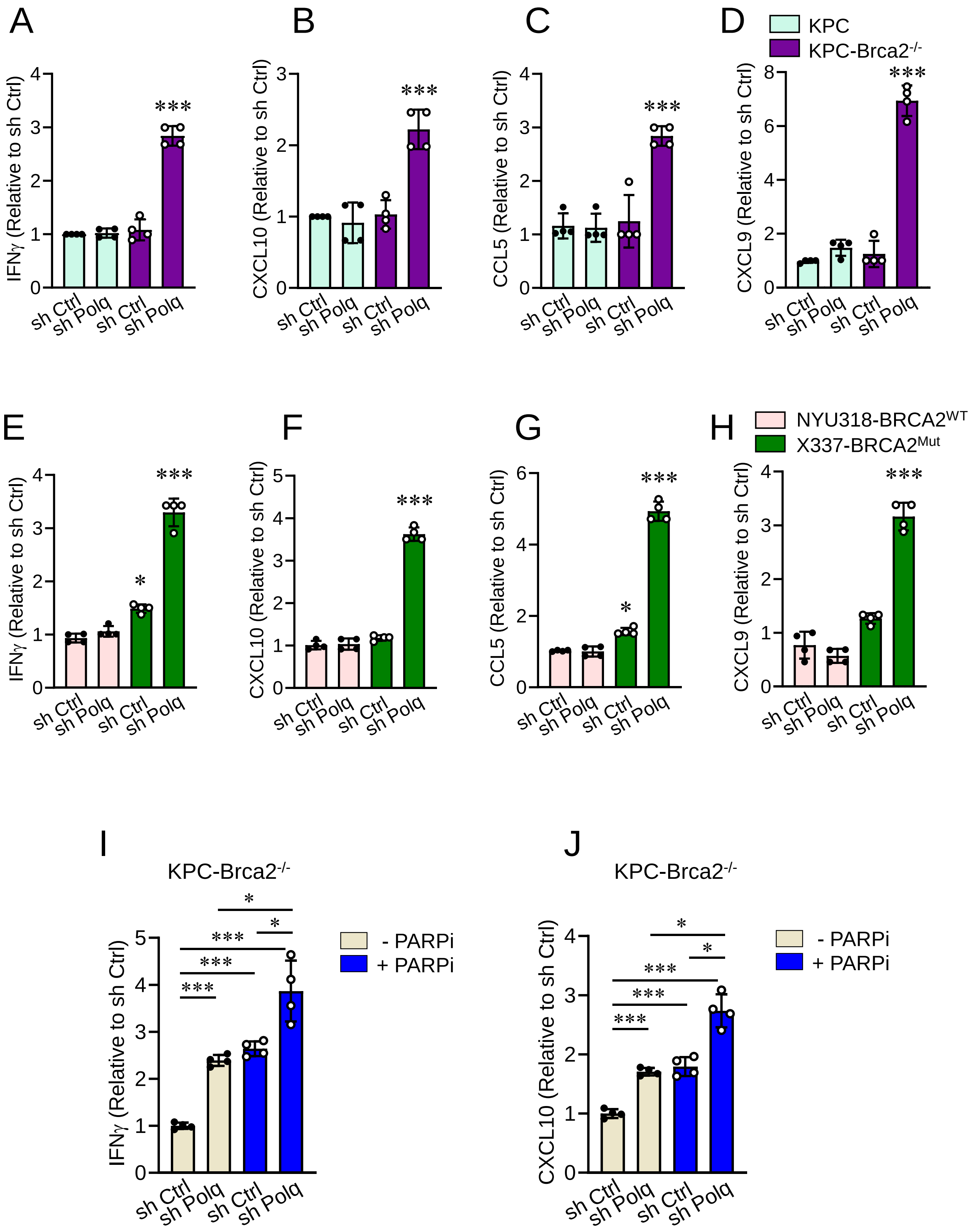

Supplemental Figure 5. POLQ inhibition enhances immune infiltration in BRCA2-deficient PDAC

(A-D) Normalized levels of immune activity markers INFγ (A), CXCL10 (B), CCL5 (C), and CXCL9 (D) in KPC and KPC-*Brca2*$^{-/-}$ sh Ctrl and sh POLQ cells as measured by cytokine array (n=4). (E-H) Normalized levels of immune activity markers INFγ (E), CXCL10 (F), CCL5 (G), and CXCL9 (H) in NYU318-*BRCA2*$^{WT}$ and X337-*BRCA2*$^{Mut}$ sh Ctrl and sh POLQ cells as measured by cytokine array (n=4). (I, J) Normalized levels of immune activity markers INFγ (I) and CXCL10 (J) in KPC and KPC-*Brca2*$^{-/-}$ cells with or without sh POLQ in combination with 2 μM PARPi (Olaparib) as measured by cytokine array (n=4). (*) $P < 0.05$, (**) $P < 0.01$, (***) $P < 0.005$), (ns) not significant. Error bars=Mean ± SEM.

Supplemental Figure S6, related to figure 6

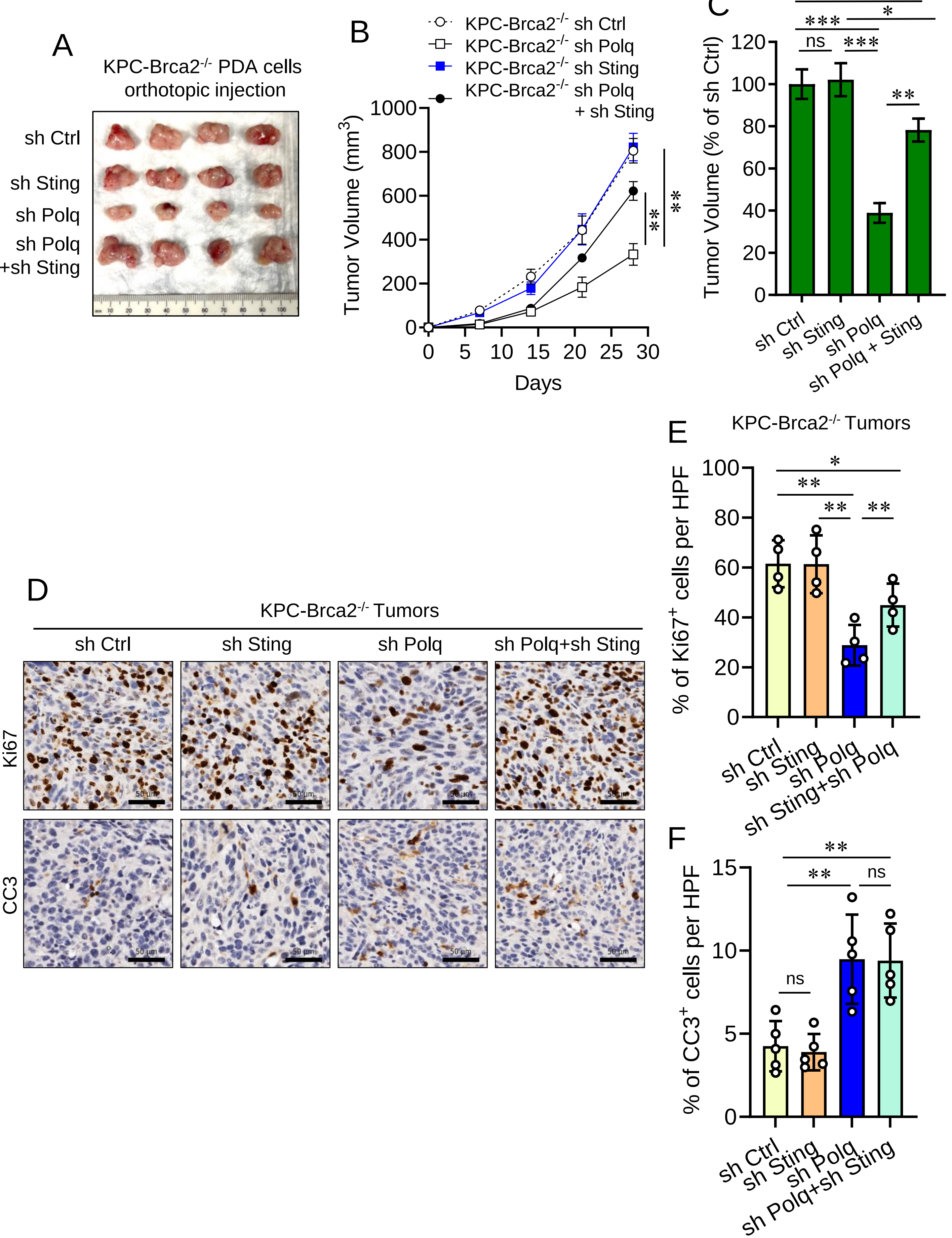

# Supplemental Figure S6, related to figure 6

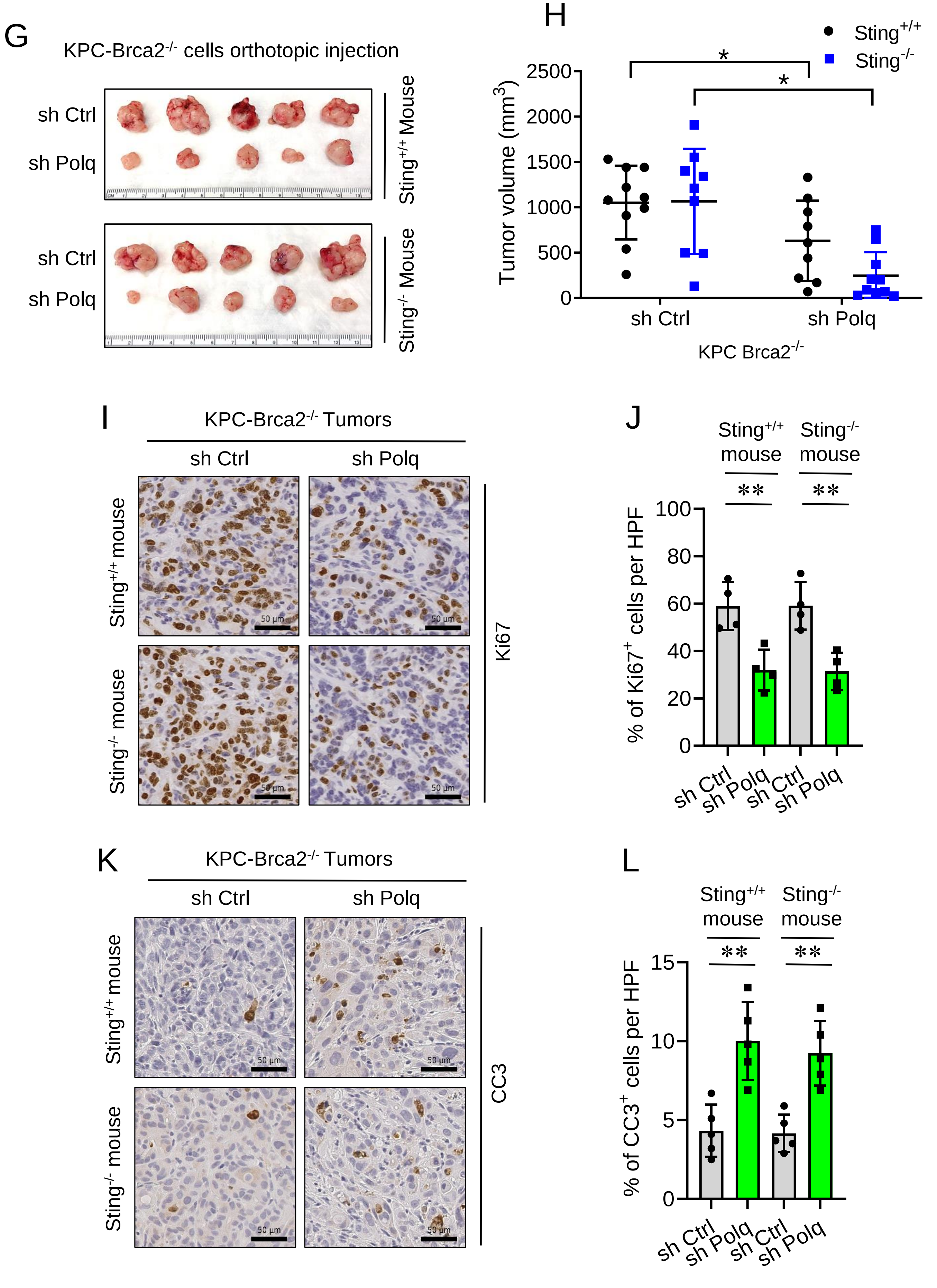

Supplemental Figure S6, related to figure 6

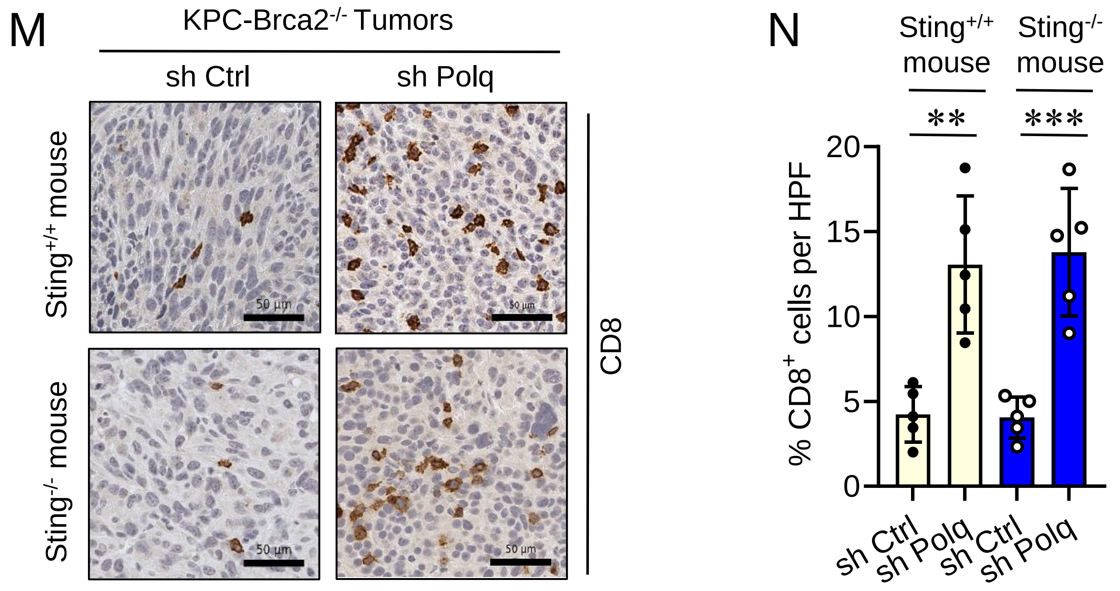


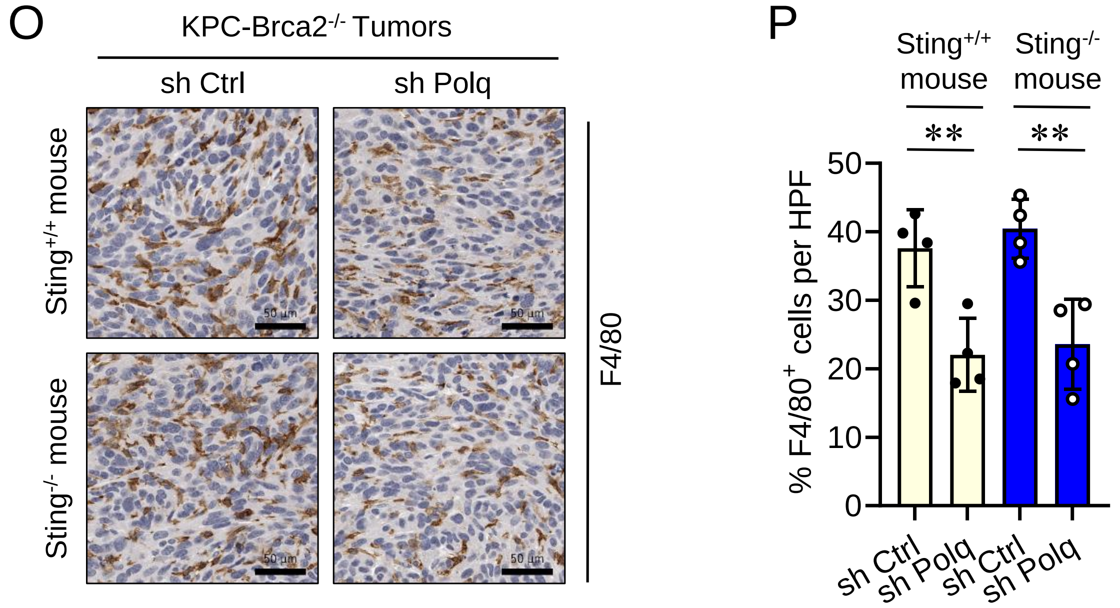


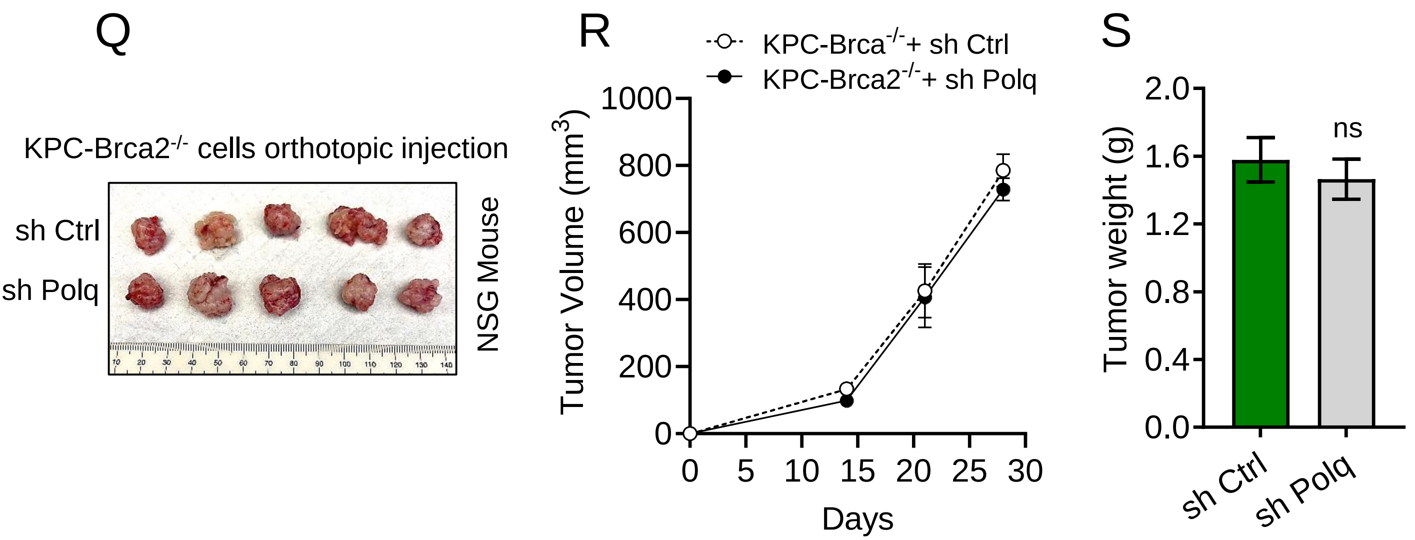

Supplemental Figure S6, related to figure 6

T

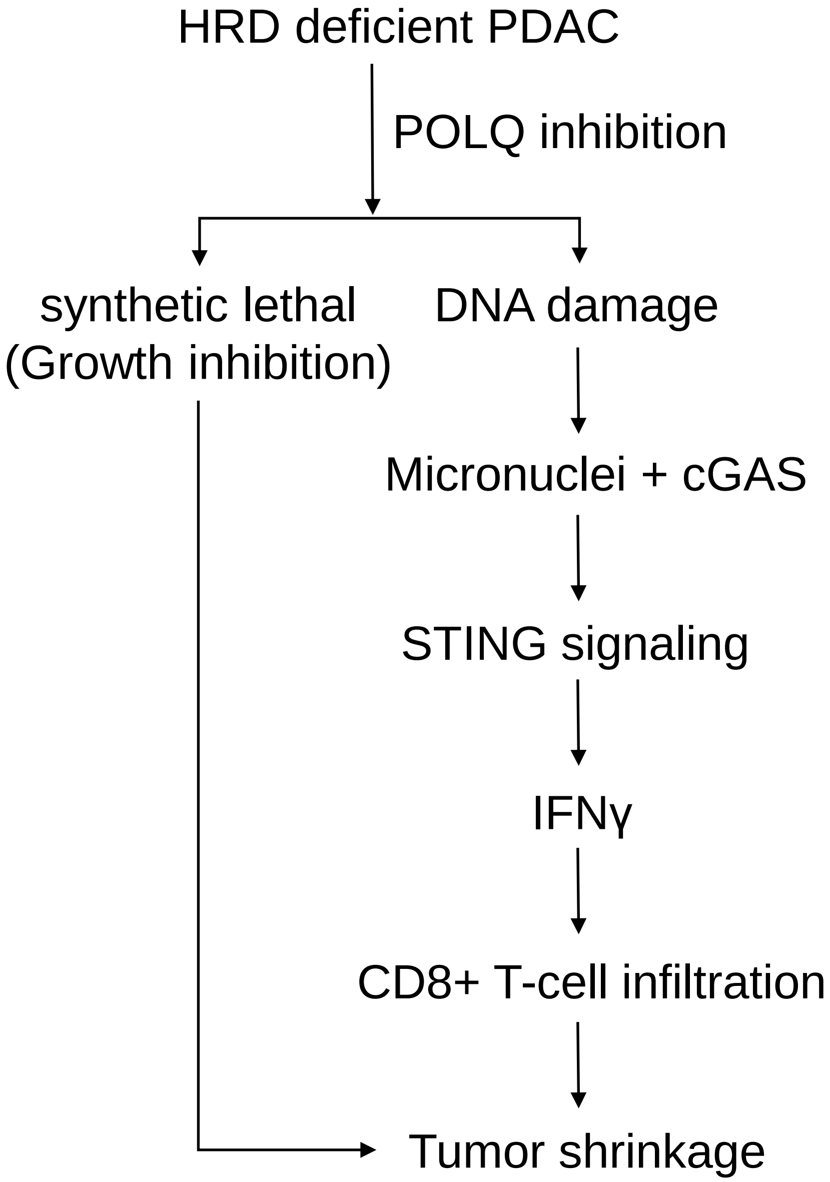

Supplemental Figure 6. STING activation is essential for the POLQ inhibition-induced antitumor effect in BRCA2-deficient PDAC. (A) Representative image of KPC-*Brca2*$^{-/-}$ sh Ctrl, sh *STING*, sh*POLQ*, and sh *POLQ* + sh *STING* tumors collected 4 weeks after orthotopic implantation into immunocompetent mice. (B) The growth curve of KPC and KPC-*Brca2*$^{-/-}$ tumors with or without sh Ctrl, sh *Polq*, sh *Sting*, or sh *Polq* + sh *Sting* (n=10/group). (C) % of Volume for KPC-*Brca2*$^{-/-}$ sh Ctrl, sh *STING*, sh *POLQ*, and sh *POLQ* + sh *STING* tumors vs sh Ctrl at 4 weeks after orthotopic implantation (n=6-8 mice/group). (D) Representative images of KPC-*Brca2*$^{-/-}$ sh Ctrl, sh *STING*, sh *POLQ*, and sh *POLQ* + sh *STING* tumors, stained for Ki67 and CC3 expression by IHC. Scale bar, 50 µm. (E) Quantification of Ki67 expression from (D). Each point on the graph represents one visual field. (F) Quantification of CC3 expression from (D). (G) Representative image of KPC-*Brca2*$^{-/-}$ sh Ctrl and sh *POLQ* tumors, collected 3 weeks after orthotopic implantation into *Sting*$^{+/+}$ and *Sting*$^{-/-}$ mice. (H) Tumor volume was measured by ultrasound in mice 3 weeks after orthotopic implantation of cells from (G) (n=9-10 mice/group). (I) Representative images of KPC-*Brca2*$^{-/-}$ sh Ctrl and sh POLQ tumors in *Sting*$^{+/+}$ and *Sting*-/- mice stained for Ki67 by IHC. Scale bar, 50 µm. (J) Quantification of Ki67+ cells per HPF from (I). (K) Representative images of KPC-*Brca2*$^{-/-}$ sh Ctrl and sh *POLQ* tumors in *Sting*+$^{/+}$ and *Sting*$^{-/-}$ mice stained for CC3 by IHC. Scale bar, 50 µm. (L) Quantification of CC3+ cells per HPF from (K). (M) Representative images of KPC-*Brca2*$^{-/-}$ sh Ctrl and sh *POLQ* tumors in *Sting*$^{+/+}$ and *Sting*$^{-/-}$ mice stained for CD8+ cells by IHC. Scale bar, 50 µm. (N) Quantification of CD8+ cells per HPF from (M). (O) Representative images of KPC-*Brca2*$^{-/-}$ sh Ctrl and sh *POLQ* tumors in *Sting*$^{+/+}$ and *Sting*$^{-/-}$ mice stained for F4/80+ cells by IHC. Scale bar, 50 µm. (P) Quantification of F4/80+ cells per HPF from (O). (Q) Representative image of KPC-*Brca2*$^{-/-}$ sh Ctrl and sh *POLQ* tumors, collected 3 weeks after orthotopic implantation into immunodeficient NSG mice. (R) The growth curve of KPC and KPC-*Brca2*$^{-/-}$ tumors with or without sh Ctrl or sh *Polq* in NSG mice (n=6-8/group). (S) Tumor volume was measured by ultrasound in C57BL/6 mice orthotopically implanted with KPC and KPC-*Brca2*$^{-/-}$ sh Ctrl and sh *POLQ* cells (n=6-8 mice/group). (T) POLQ inhibition in HR-deficient PDAC promotes tumor cell death by 1) synthetic lethal interaction and 2) recruitment of immune cells through the cGAS-STING pathway.

Data are representative of at least three independent experiments. (*) P < 0.05, (**) P < 0.01, (***) P < 0.005), (ns) not significant. Bars are the mean ± SEM.
