# Supplementary material for: POLQ inhibition elicits an immune response in homologous recombination–deficient pancreatic adenocarcinoma via cGAS/STING signaling
Source: J Clin Invest. 2023 Jun 1;133(11):e165934. doi: 10.1172/JCI165934 (PMC10232002; doi:10.1172/JCI165934)
Supplement: Supplemental table 1 [file jci-133-165934-s221.pdf]

Supplemental Table 1. Primary Antibodies list

Antibody	Company	Catalog number	Host species	Application
cGAS (human-specific)	Cell Signaling Technologies	79978s	Rabbit	WB, IF
cGAS (mouse-specific)	Cell Signaling Technologies	31659s	Rabbit	WB, IF
STING	Cell Signaling Technologies	50494s	Rabbit	WB, IF
p-TBK	Cell Signaling Technologies	5483s	Rabbit	IF
γH2AX	Cell Signaling Technologies	7631s	Rabbit	IF, IHC
Rad51	abcam	ab133534	Rabbit	IF
Ki67	Cell Signaling Technologies	9129s	Rabbit	IHC
Caspase-3 (CC3)	Cell Signaling Technologies	9664s	Rabbit	IHC
F4/80	Cell Signaling Technologies	29047s	Rabbit	IHC
CD4	abcam	ab182685	Rabbit	IHC
CD8	abcam	ab209775	Rabbit	IHC
Granzyme B	abcam	ab4059	Rabbit	IF
CD8	Invitrogen	MA1-10301	Rat	IF

IF, immunofluorescence; IHC, immunohistochemistry; WB, Western blot.
